# Modeling the Underlying Dynamics of the Spread of Crime

**DOI:** 10.1371/journal.pone.0088923

**Published:** 2014-04-02

**Authors:** David McMillon, Carl P. Simon, Jeffrey Morenoff

**Affiliations:** 1 Department of Mathematics and Department of Industrial and Operations Engineering, University of Michigan, Ann Arbor, Michigan, United States of America; 2 Department of Mathematics, Center for the Study of Complex Systems, and Gerald R. Ford School of Public Policy, University of Michigan, Ann Arbor, Michigan, United States of America; 3 Department of Sociology, Institute for Social Research, Population Studies Center, University of Michigan, Ann Arbor, Michigan, United States of America; University of Maribor, Slovenia

## Abstract

The spread of crime is a complex, dynamic process that calls for a systems level approach. Here, we build and analyze a series of dynamical systems models of the spread of crime, imprisonment and recidivism, using only abstract transition parameters. To find the general patterns among these parameters—patterns that are independent of the underlying particulars—we compute analytic expressions for the equilibria and for the tipping points between high-crime and low-crime equilibria in these models. We use these expressions to examine, in particular, the effects of longer prison terms and of increased incarceration rates on the prevalence of crime, with a follow-up analysis on the effects of a Three-Strike Policy.

## Introduction

The U.S. spends more on criminal justice than any other nation, investing approximately eighty billion dollars per year on state and federal prisons that currently hold 2.24 million Americans [1], [2]. In the last two decades, state spending on prisons grew at six times the rate of state spending on higher education [2]. One study [3] found that an average of 6.8 percent of state general funds in 2007 went to corrections agencies, making corrections the fifth-largest state budget category (behind health, elementary and secondary education, higher education and transportation). Moreover, the rise in incarceration in the United States over the past three decades has been experienced disproportionately by minorities, particularly young black men, and those with low levels of education [3]. Over half of African-American men with less than a high school degree go to prison at some time in their lives [4], and African-American males make up a third of America's incarcerated population [5]. Many metaphors have been offered to characterize the resulting system of criminal punishment, such as the “era of mass imprisonment” [6] [7], the “New Jim Crow” [8], and the “Cradle-to-Prison Pipeline” [5].

In this paper we develop simplified models that capture this pipeline as a series of flows into and out of states of criminal activity, incarceration, and recidivism within a population. By analyzing the resulting system of equations, we derive the low- and high-crime equilibria to which these flows lead and the threshold points that “tip” the system toward one of these equilibrium points. We also demonstrate how policies that manipulate key parameters in the system – e.g., by affecting the rate at which people are incarcerated and the rate at which they are released from prison/jail – ultimately affect the proportion of criminally active people in the population. The results illuminate the social conditions under which marginal increases in the rate of incarceration will augment or diminish the spread of crime. The models presented in this paper also lay a foundation for future work that will incorporate empirical estimates to trace the system-wide implications of changes in criminal activity and incarceration.

### Mathematical Models of Crime and Punishment

We build on a tradition of systems models applied to crime that began with the seminal work of the Science and Technology Task Force of the President's Commission on Law Enforcement and Administration of Justice [9]. This work demonstrated how systems models could be used to project the workload and operating costs of police, courts, and corrections, and analyze the effects on crime rates and criminal justice costs of changes to the system initiated by policy (e.g., increasing the size of the police force) or demographic shifts in the population (e.g., the baby boom). Following the Commission's report, operations researchers qua criminologists developed more sophisticated mathematical models to capture “the feedback into society of offenders released at various stages in the system” [10], with a focus on modeling the recidivism process [11], [12], [13], [14], [15], [16]. For example, Blumstein [17] and others used such models to identify minimum cost methods to reduce crime through the strategic increase and placement of police forces and to advise policy-makers on the effects of incarceration policies based on estimates of the crimes averted by incarcerating criminally active offenders.

Over time, the focus of this work turned away from macro-level models of crime rates and feedback processes to micro-level analyses of so-called criminal careers and how offending is affected by punishment [17]. In the early research on criminal careers, Blumstein and colleagues [18],[19] analyzed trajectories of individual participation in crime, beginning at the age of “initiation” and continuing until “desistance,” estimating the individual-level rate of offending (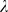) and how it changed over time. Some scholars argued that incarceration would be maximally effective at reducing crime when reserved for individuals who would have relatively higher rates of offending were they not behind bars – a policy position that became known as “selective incapacitation” [20]. However, the idea of differentially punishing people based on their expected future behavior was heavily criticized on ethical grounds, especially given the error-prone nature of predicting one's future involvement in crime. Subsequent research revealed that even without an explicit policy aimed at selective incapacitation, the inmate population has a much higher estimated rate of offending, whereas in the population of non-incarcerated offenders the rate of offending is lower and the distribution is much more heterogeneous [21].

Another line of research focused on sanction policies that reduce crime through general or specific deterrence [22],[23]. Durlauf and Nagin [24] recently reviewed this research and concluded evidence that incarceration deters crime is weak. Aggregate studies show that increases to prison sentence lengths are associated with weak to modest declines in crime, while micro-level studies suggest that experiencing incarceration does not seem to prevent reoffending. The most substantial deterrent effects, in their estimation, come from implementing tactics that increase the perceived risk of apprehension.

Another relevant area of research inspired by systems perspectives on crime assesses the extent to which some crimes spread through a process of diffusion or contagion. For example, Blumstein [25] argued that a major reason why youth violent crime rates rose dramatically during the 1980s was due to a diffusion process set off by the introduction of crack cocaine in many American cities. The considerable profit margins that could be gained from selling crack led many participants in illicit drug markets to arm themselves for self-protection, setting off an “arms race” – even among neighborhood residents not connected to the drug market – and leading to more gun-related violence that spread in a diffusion-like process to surrounding neighborhoods [26]. Blumstein [27] also suggested the increasing incarceration rates for drug offenders contributed to the diffusion of crime by necessitating the recruitment of more young people into crack markets and gun-related violence.

A related tradition of work on mathematical models of crime stems from Becker's treatment of crime as a rational decision-making process in which the individual compares the benefits and costs (punishment) associated with criminal activities against alternatives to crime [28]. For example, Freedman and colleagues [29] developed a model explaining how crime becomes concentrated in certain neighborhoods, where the expected monetary return from committing a crime (the probability of not being arrested times the reward of the crime) exceeds the opportunity costs for crime. Wang et al [30] generalized this approach – by allowing opportunity costs to be heterogeneous across potential criminals and depend on the level of crime in a particular neighborhood – and derived the equilibrium amount of criminal activity in a neighborhood.

In another approach to the spatial dynamics of crime, Short and colleagues [31] developed an agent-based model in which the risk of a site becoming a target of a burglary is a function of past burglaries at the site and in neighboring locations; they determine the parameter values that lead to the emergence of stable hotspots. Building on this paper, Short and colleagues [32] used reaction-diffusion partial differential equations to show that hotspots can emerge, as either supercritical or subcritical bifurcations, when diffusion enhances the risk of repeated crimes in a local area. Other papers that have contributed to this line of work include one [33] that proved the existence and uniqueness of the solutions to the coupled system of partial differential equations presented by Short et al [31], and another [34] showing that Short et al's system [31] supports global bifurcation of spatially varying solutions (i.e., hot spots) from a spatially constant equilibrium. Short et al [35] bring some empirical data to such victimization studies when they fit 2000–2005 home burglary data from Long Beach, California to probability distributions of the time intervals between return trips of burglars to Long Beach homes.

Yet another approach to modeling crime mathematically has been taken by scholars applying game theoretic models based on the classic prisoner's dilemma paradigm and its variants, in which players choose to cooperate or defect in their interactions with each other. Some papers, such as [36] and [37], model defectors as bringing harm to cooperators and include a role for “punishers” who go out of their way to punish defectors. Research studies [36] and [37] include informants, who act as defectors but also inform on other defectors; [36] uses replicator dynamics of game theory, while [37] relies on behavioral lab experiments to show that informants play a key role in possibly moving the population to a crime-free long-run equilibrium. Jiang et al [38] use the related snowdrift game in which they allow cooperators to levy fines on defectors. They use behavioral lab experiments to argue that smaller fines work better when cooperation is more likely. Perc et al [39] use a two-dimensional, four-parameter inspection game that includes criminals, noncriminals, and punishing inspectors as players. Including a cost of detecting crime and a fine for detected criminals, they set up an evolutionary game dynamic and use Monte Carlo simulations and agent-based models to describe how the outcomes change as the underlying parameters cross various thresholds. They show, for example, that increasing punishment need not decrease crime. The punishment regimes in all these papers are peer-to-peer.

Berenji et al [40] use an evolutionary game theoretic model to study the hypothetical effects of incarceration and prisoner reentry policy interventions on recidivism. In their simulation exercise, agents repeatedly decide whether to reform or continue engaging in criminal activity, and the outcome can be a society with a majority of “virtuous, rehabilitated citizens” or “incorrigibles,” depending on the value of the parameters driving decision-making. They find that excessively harsh or lenient punishments are both less effective at reducing crime than a policy that optimally dedicates scarce resources to a mix of both punishment and post-punishment intervention programs, especially to offenders returning from prison for the first time. As did [36], they formulate an approximate system of ordinary differential equations (ODEs) that yield similar results, via Monte Carlo simulations.

In this paper we take a population-based approach, similar to some of the systems models and game theoretic models surveyed above, but rooted in models that have been developed mainly to model the spread of disease. Population-based studies of the spread of disease have led to major breakthroughs in our understanding of disease spread. Disease models have been successfully used to forecast the onset and spread of worldwide influenza epidemics and to design vaccination programs for childhood diseases like measles and rubella. Sir Richard Ross's 1911 malaria model introduced the key concept of a threshold for epidemic spread and used it to show that malaria could be controlled without killing every mosquito in the infected area [41], [42], [43]. We will compute and analyze similar thresholds, considering the spread of crime and the dynamics of incarceration. In the interest of offering a relatively parsimonious treatment of the population dynamics of crime and punishment, the models presented below assume homogeneous populations, do not take stock of differences by age, frequency of offending, crime type, or actions taken at different stages of the criminal justice system (e.g., arrest, conviction, sentencing, parole). We have also not yet included empirical data. We intend to address each of these limitations in future work. At present, we seek to gain as much mathematical information as we can from the basic foundational models presented in this paper.

### Overview of Models and Analytic Strategy

In this paper we present a progression of increasingly complex models that capture population flows into and out of states that differentiate segments of the population based on participation in crime and incarceration. We first explain the general framework for all of these models and then describe how the models are differentiated based on their increasing complexity.

**Figure 1:**
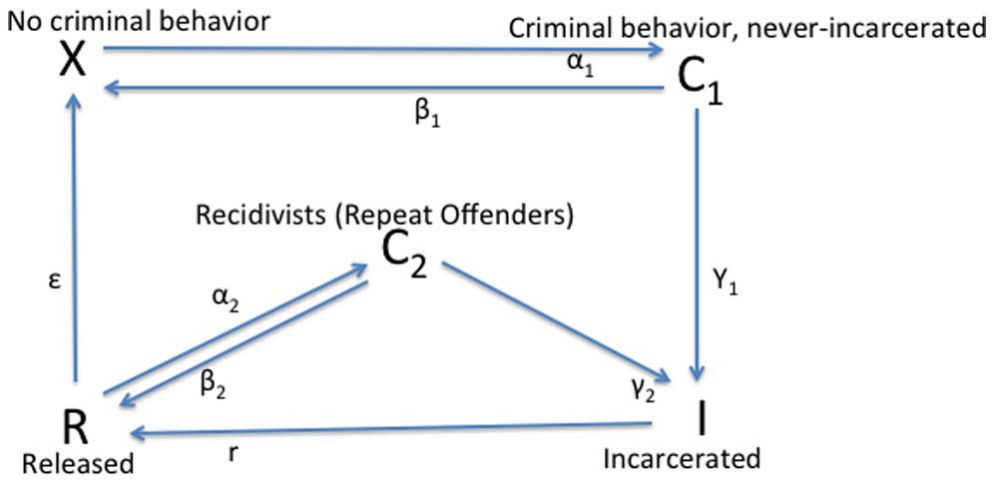
Flow Diagram for the Main Model.

The fundamental components of our models are presented in Figure 1, which illustrates the flows between five states of criminal activity: (1) 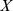, those who are not criminally active at a given time; (2) 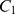, those who are criminally active but have never been incarcerated; (3) 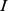, those who are incarcerated at a given time; (4) 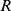, those who were once incarcerated but are not criminally active; and (5) 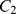, those who were once incarcerated and are again criminally active. (Note that we define repeat offenders based on the number of times they have been incarcerated. This is different from the previous approach of [11] which used arrests as the basis for defining repeat offenders.)

In these models we take on a very high-level perspective of criminal dynamics. This perspective, unlike earlier models, allows us to investigate simultaneously the systematic effects of contagion, desistance, incarceration rates of first-time and repeat-offenders, prison term length, and criminal rehabilitation/redemption on long-run crime and incarceration outcomes. Unlike earlier models, such as [10], [11], [12], these models do not differentiate between actions taken at different stages of the criminal justice system, such as arrest, conviction, sentencing, and community supervision (although we intend to develop more complex models that account for these stages in future work). We also do not differentiate between offenders based on the frequency of their activity (i.e., individual crime occurrences).

**Figure 2:**
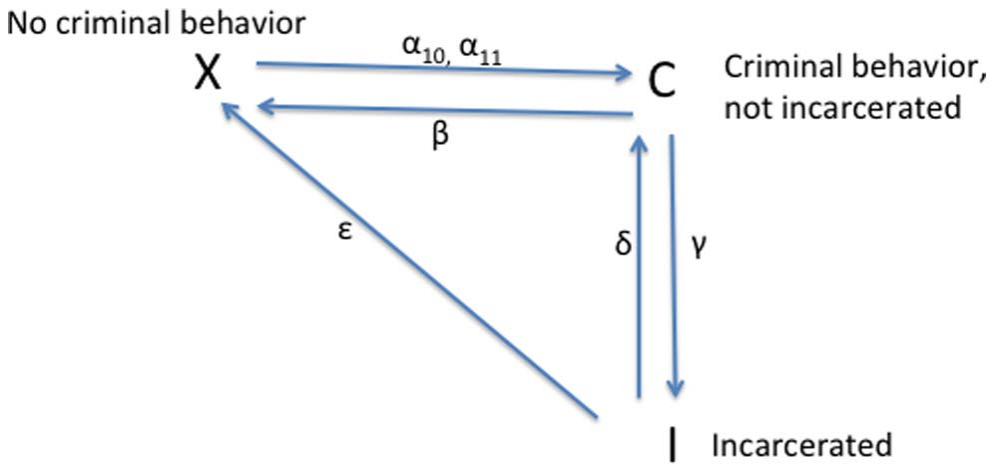
Flow Diagram for Models 1 and 2.

With these caveats in mind, we can use models such as the one depicted in Figure 1 to parameterize the following types of population flows.

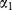
 The rate at which individuals move from state 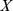 (not criminally active) to 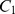 (criminally-active but not incarcerated). This can be thought of as the rate of onset, or initial participation in crime. In subsequent models we decompose 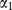 into two separate paths (see Figure 2, for example). The first, 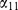, represents the flow into criminal activity that depends on having contact with other criminally active people. It is analogous to the “effective contact rate” in infectious disease models, an indicator of the extent to which having more contact with criminally-active people increases one's risk of participating in crime, and it relates to many criminological theories premised on the idea that patterns of social interactions influence decisions to participate in crime. For example, social learning theory views social interactions as platforms for learning information relevant to crime; labeling theory emphasizes the importance of interactions for forging criminal identities; and subcultural theories focus on how youth can be drawn into criminal activity through peer interactions in the context of groups or neighborhoods with strong social norms emphasizing toughness and violence as a means of resolving conflict [44]. The second pathway, 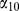, represents transitions into criminal activity that are not dependent on having contact with criminally-active people, but are instead driven by individual differences in the propensity toward crime [45], [46].

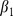
 The rate at which individuals move from state 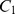 (criminally-active but not incarcerated) to 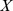 (not criminally active). This parameter represents a desistance process that is not related to the experience of incarceration. Thus, it could represent the effect of general deterrence but not specific deterrence (at least not the specific experience of incarceration) or rehabilitation (at least not from treatment received during or after incarceration). For example, it could be related to social interventions that aim to reform young criminals who have not yet been incarcerated. It could also be influenced by many other factors, including aging out of crime.

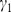
 The rate at which individuals move from state 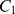 (criminally-active but not incarcerated) to 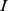 (incarcerated). This parameter represents the rate at which criminally-active individuals are incarcerated. In a sense, it combines processes related to the police, courts, and correctional systems.

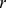
 The rate at which individuals move from state 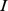 (incarcerated) to 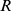 (formerly incarcerated but not criminally-active). This parameter is the rate at which individuals are released from prison/jail, and 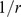 represents the average length of a prison/jail term. We note that some individuals may resume criminal activity very soon after being released from prison or jail, but the model assumes that everyone returning from prison/jail is initially (even if only for a matter of minutes, hours, or days) not active in crime.

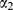
 The rate at which individuals move from state 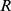 (formerly incarcerated but not criminally-active) to 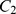 (formerly incarcerated and criminally-active). This parameter captures recidivism. It is the rate at which people resume criminal activity after their release from prison or jail. In later models, we decompose 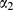 into 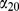 and 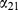 as we did for 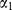.

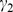
 The rate at which individuals move from state 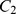 (formerly incarcerated and criminally-active) to 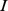 (incarcerated). This parameter captures the second component of the recidivism process – the rate at which former offenders who are criminally-active are incarcerated. Thus, 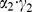 represents the rate at which formerly incarcerated individuals are returned to prison/jail.

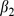
 The rate at which individuals move from state 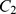 (formerly incarcerated and criminally-active) to 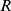 (formerly incarcerated but not criminally-active). This parameter represents the desistance from crime among those who have been incarcerated. Thus, unlike 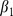, 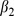 can be influenced by specific deterrence and rehabilitation.

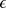
 The rate at which individuals move from state 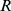 (formerly incarcerated but not criminally-active) to 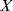 (not criminally active). Like 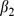, 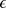 represents a desistance process among formerly incarcerated individuals. The key difference between 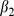 and 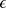 lies in the states to which they lead. 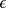 leads back to 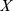, which means that it captures pathways among people who are, for all intents and purposes, treated as “non-criminals.” In some cases, this could result from having one's felony record formally expunged. It can also capture what Blumstein and Nakamura [47] call “redemption” in reference to “the process of ‘going straight’ and being released from bearing the mark of crime.”

The models we present in this paper represent increasingly complex depictions of these processes along the two dimensions depicted in Table 1. One way to differentiate the complexity of the models is by how many different compartments or ‘dimensions’ they represent. In the simplest models (1 and 2), we include only three compartments: not criminally active (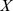), criminally-active (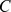), and incarcerated (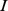). The five-dimensional models (3 and 4) capture the process of prisoner reentry (or returning from jail) by introducing the distinction between states 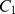 and 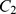 (based on whether one has been incarcerated before) and introducing state 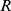 to represent the state one is in immediately after release from prison/jail. The nine-dimensional models (5 and 6) introduce temporal distinctions between different spells of incarceration in order to analyze the effects of a three-strikes policy. These models differentiate between 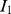, 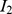, and 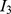 (the first, second, and third or higher spell of incarceration), and between 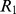 (returning from a first spell of incarceration) and 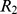 (returning from a second spell of incarceration).

**Table 1:** Classification of Our Models.

Flow into Criminal Activity	3 Population States	5 Population States	9 Population States
Only Contagion	Model 1	Model 3	Model 5
Contagion, Individual Propensity	Model 2	Model 4	Model 6

Another aspect of the complexity of these models is the way that they account for the transition to criminal activity. In the odd numbered models, the only way someone can become criminally-active is to interact with another person who is already criminally-active. This restriction is necessary in order to illustrate the dynamics of a system that would lead to a “crime-free” equilibrium. Put differently, if new criminals could only emerge by interacting with existing criminals, then diminishing the number of criminally-active people (either through incarceration or desistance) could lead to the full eradication of crime. Although not a realistic possibility, understanding the conditions that lead to such crime-free equilibria is a necessary step in deriving our analytic expressions for the low-crime/high-crime threshold and high-crime prevalence in the more general models.

For each of these models we compute an explicit expression for the tipping point or threshold between the crime-free (or low-crime) equilibrium and the high-crime endemic equilibrium, analogous to the basic reproduction number 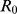 in epidemiology and demography. For many of them we also compute an explicit expression for the prevalence of criminal activity at the high-crime equilibrium. We use these expressions, especially for our main five-dimensional model, to demonstrate how policies that manipulate key parameters in the system – e.g., by affecting the rate at which people are incarcerated and the rate at which they are released from prison/jail – ultimately affect the proportion of criminally active people in the population. The results illuminate the social conditions under which marginal increases in the rate of incarceration will augment or diminish the spread of crime.

*At this point, readers more interested in the bottom line than the mathematical analyses and model building may wish to skip to the* *Results and Discussion* *Section, which discusses policy implications that emerge from our mathematical analyses.*

## Models and Analyses

### Model 1: Simplified 3-dimensional Model

The model diagramed in Figure 1 is a five-dimensional dynamical system – a challenging system to analyze. To gain some intuition for the underlying dynamics of this system, we construct and analyze the simplest possible three-dimensional system that retains the key features of the 5D system but whose reduction to two dimensions can be studied analytically and geometrically as a planar system. This three-dimensional model, Model 1, has only the three basic compartments: the non-criminally-active 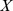, the criminally active 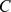, and the incarcerated population 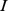. If one ignores the dotted 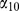 arrow, Figure 2 presents this model's compartmental diagram. The parameters are summarized below, followed by the associated system of ordinary differential equations.

#### Parameters for Models 1 and 2


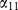
 Contagion parameter of criminal behavior.


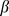
 Rate at which criminals discontinue criminal habits (desistance).


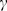
 Rate at which criminals are incarcerated.


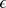
 Rate at which incarcerated individuals are released and assimilate back into society.


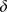
 Rate at which incarcerated are released and return to criminal life.
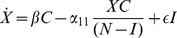
(1a)

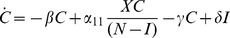
(1b)

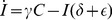
(1c)

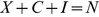
(1d)


Equation (1a) represents the dynamics of the law-abiding population 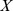. The first term 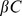 represents the flow from 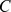 back into 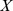, in which criminals discontinue criminal habits, whether through social interventions or fear of incarceration. The second term 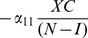 captures the flow into the criminal population 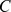 due to interactions with criminals. The 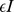 term captures the dynamic in which former criminals released from incarceration are able to assimilate back into society.

Non-criminally active citizens in equation (1a) become criminally active only through interactions with active criminals, an assumption that leads to the possibility of a crime-free equilibrium. For Model 1's crime-free equilibrium we derive threshold conditions among the model parameters. We also compute an explicit formula for the “endemic” high-crime equilibrium. Model 2 will include the possibility that law-abiding citizens can turn to crime without the “contagion effect” of interactions with active criminals – an assumption that precludes a crime-free equilibrium. Without a crime-free equilibrium, that is, without zero as a reference point, Model 2 is more difficult to analyze; but we use Model 1 to derive conditions that separate convergence to a low-crime equilibrium from convergence to a high-crime equilibrium in Model 2.

Equation (1b) represents the changes within the criminal population 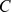, either via reform (first term), new recruits from 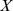 (second term), incarceration (third term), or recidivism after incarceration (fourth term). Equation (1c) captures the changes within the incarcerated population: flow into 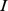 from 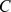 (first term) and flow out to 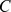 or 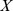 (last terms) after release.

Since the sum of the right hand sides of these three equations adds to zero, 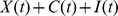 is constant over time and equal to the size of the total population 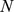, as indicated in Equation (1d). Consequently, we can treat the above system as a system of two differential equations in two unknowns. We choose to replace 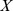 in Equation (1b) by 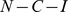 and work with a system (2) of two equations involving only the variables 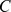 and 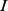.
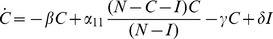
(2a)

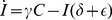
(2b)


We will analyze the equilibria of this two-dimensional system in three different ways: 1) by studying its two-dimensional geometric phase portrait to separate convergence to high-crime equilibrium from convergence to low-crime equilibrium, 2) by explicitly computing its endemic (“high-crime”) equilibrium, and 3) by using a Lyapunov function to find the tipping point that distinguishes convergence to high-crime equilibrium from convergence to low-crime equilibrium. The first path is only possible for this two-dimensional case, but it helps us carry out paths 2) and 3) for the higher dimensional cases.

#### Phase Plane Analysis of System (2)

**Figure 3:**
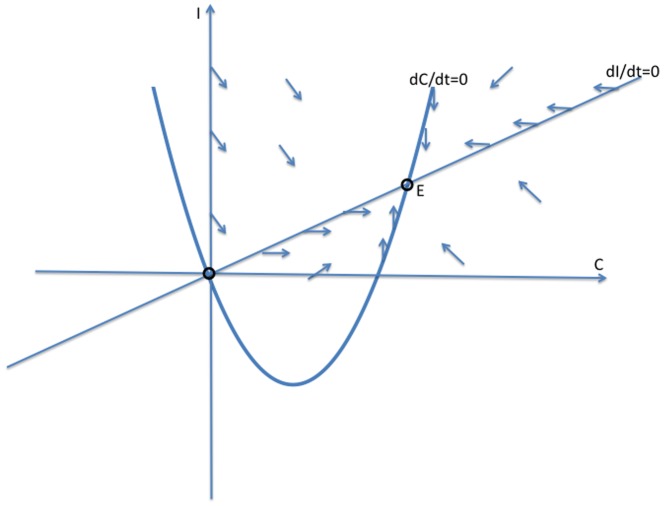
Case 1 in the study of System (2).

**Figure 4:**
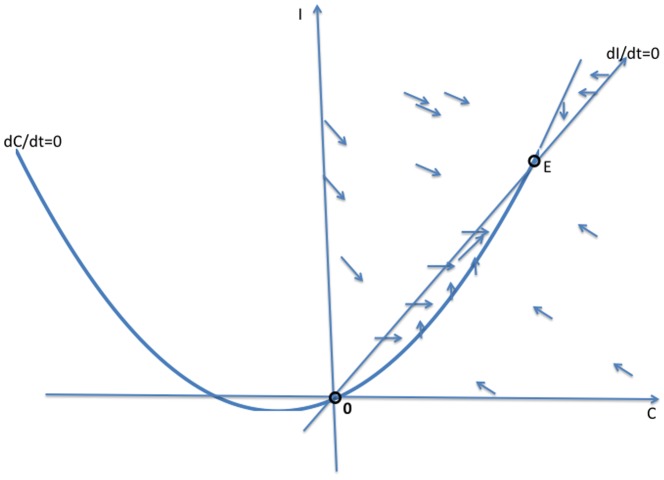
Case 2 in the study of System (2).

**Figure 5:**
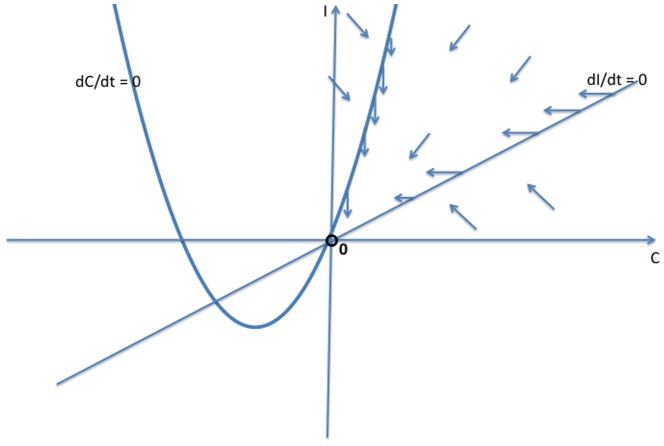
Case 3 in the study of System (2).

In this section, we solve the reduced two-dimensional dynamical system (2) geometrically, as illustrated in Figures 3, 4 and 5. We begin with the 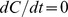 and 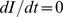 isoclines where the vector field defined by this system (2) is horizontal and vertical, respectively, in the 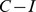 plane. The arrows of the dynamics in the regions between these isoclines indicate the flow toward an equilibrium. Note from (2b) that the 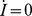 isocline in a line through the origin with slope 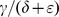, while from (2a) the 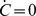 isocline is a concave-up quadratic curve with zeros in the 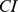 plane at
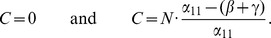



There are three cases to consider for the relative arrangement of the 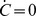 and 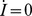 isoclines, which we label as 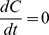 and 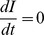 in the following figures.

**Case 1:** 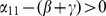. Both intercepts of the curve 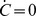 lie on the non-negative 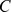-axis, and the isoclines intersect each other twice as in Figure 3. As indicated by the vector field in Figure 3, the origin **0** is an unstable steady-state, and all orbits go to the endemic equilibrium **E**.

**Case 2:** 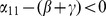 and 
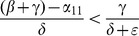
. The second zero of 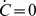 is negative and the slope of 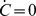 at the origin is less than the slope of 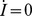 at the origin. It follows that the quadratic curve crosses the 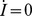 line from below and therefore crosses it again in the positive quadrant. As indicated by the vector field in Figure 4, the origin 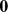 is an unstable steady-state, while the endemic equilibrium 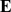 is globally stable.

**Case 3:** 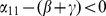 and 
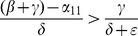
. In this case, the second zero of 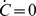 is negative and the slope of 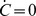 at the origin is greater than than the slope of 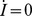 at the origin. It follows that the curve lies above the 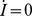 in the positive quadrant. The crime-free equilibrium 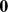 is the only steady-state in the nonnegative quadrant and, as we see with the vector field drawn in Figure 5, 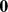 is globally stable in that quadrant.

In summary, when the second 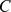-intercept of the 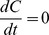 isocline is negative, i.e., 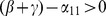, and the slope of the 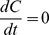 isocline is steeper than the slope of the 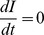 line at the origin, as in Figure 5,
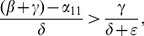
(3)then, the system converges to the crime-free equilibrium. Otherwise, the system tends to a high-crime endemic equilibrium. Expression (3) can also be written as
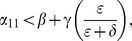
(4)or equivalently
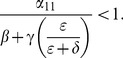
(5)


**Remark:** Strictly speaking, the dichotomy of phase portraits and the underlying behaviors do not depend on the precise functional forms in system (2), as long as the curves intersect as they do in Figures 3, 4 and 5. Of course, the analytic expressions we present do rely on the specific functional forms.

#### Threshold Condition for System (2)

Inequality (5) is the threshold that distinguishes the case in which all solutions converge to the crime-free equilibrium from the case in which all solutions converge to a high-crime equilibrium. The numerator in threshold inequality (5) is the input rate into the criminal class. The denominator is the rate out of the criminal class, with the incarceration rate 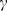 multiplied by a non-recidivism factor: 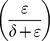. The impact of the incarceration rate 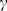 relative to the rehabilitation rate 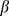 is attenuated by the fact that some of those incarcerated will return to crime. To reach a crime-free equilibrium, a community needs the contagion rate of criminal behavior 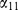 to be small relative to the success rate of social interventions for criminal desistance 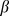 and the incarceration of criminals 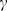.

Following terminology of the *basic reproduction number* in demography and epidemiology, we denote this ratio as 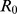. In epidemiology, 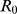 can be interpreted as the number of new infections attributed to a single infected in the course of his or her infection (in a population of susceptibles). Similarly, in our model, 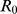 can be interpreted as the number of people a criminally active person can seduce to criminal activity during the period of his or her active criminal behavior.

#### Endemic Equilibrium in System (2)

We compute the fractions of the criminally active and the non-criminally active people “on the street” at the endemic equilibrium for system (2) Since 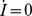 at the equilibrium, 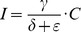 by (2b).

Since 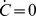 and 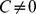, by (2a),
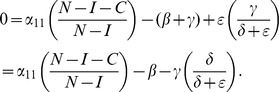
Therefore,
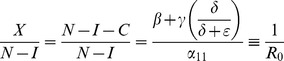
(6)and
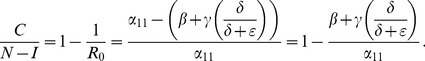
(7)the equilibrium prevalence (6) of noncriminals is simply the ratio of the rate of *leaving* a life of crime (desistance rate 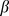 plus the weighted enforcement rate) and the rate of *entering* a life of crime (the effective contact rate 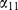).

Equations (6) and (7) are analogous to those in the simple SIS model of disease spread, where the endemic prevalence of disease is 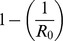.

In other words, our analytic expression (5) for the tipping point 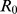 that separates convergence to a crime-free equilibrium from convergence to a high-crime endemic equilibrium can also be used to calculate the proportion of non-imprisoned individuals who are law-abiding citizens 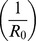 and the proportion who are criminals 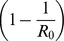.

#### Stability of the Crime-Free Equilibrium of System (2) Via Lyapunov Functions

In the previous subsection, we used a planar phase portrait of system (10) to derive the threshold for the crime-free equilibrium to be globally asymptotically stable. We cannot use this geometric approach for the Full 5D Model of Figure 1. In this subsection, we use the geometric intuition developed in the analysis of Figure 2 in the “Phase Plane Analysis of System (2)” Subsection to construct an analytic derivation of the 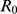 expression that will work in the Full 5D Model.

**Figure 6:**
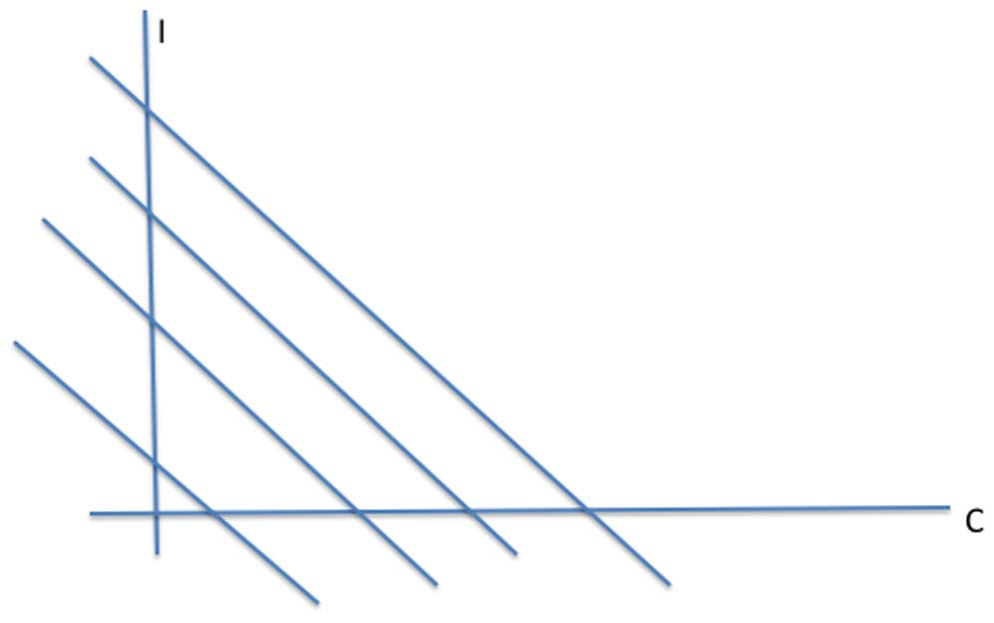
Levels sets of *V*.

As Figure 5 suggests, in the situation where the crime-free equilibrium is globally asymptotically stable, the vector field of system (2) points mostly in the direction of the origin. To prove this analytically, we will construct a linear real-valued “Lyapunov function” 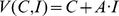, where 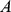 is a positive constant so that solutions of (2) move to lower and lower level sets of 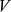 (level sets of 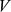 pictured in Figure 6) — that is, so that 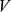 decreases along solutions 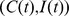 of system (2) and therefore solutions are “forced” to the origin.

We seek 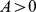 such that the derivative of 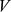 along solutions
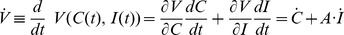
(8)is negative. We will show that such an 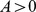 exists if and only if the conditions of Figure 5 hold, i.e., inequalities (3), (4), or (5).
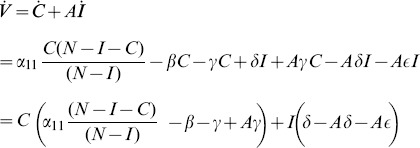



For 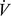 to always be negative, the two expressions in the large parentheses in the line above must be negative. However,
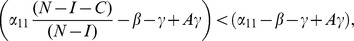
which is less than zero if and only if
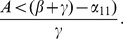



On the other hand, 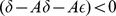 if and only if 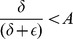. Therefore, we can construct a Lyapunov function with the desired properties as long as there exists 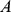 such that
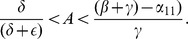



But the condition for finding such a positive constant 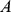 is simply that:
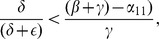
which is the same as our threshold condition (3) above.

We will use this Lyapunov function method to find the threshold for our later five- and nine-dimensional models.

### Model 2: Beyond Contagion, a More Complex 3-Dimensional Model

The first simplification that we relax is our assumption that people turn to crime only through interactions with the criminally active. We add the possibility that some non-criminals slip into crime without the influence of the criminally active, by adding 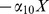 to the 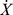 equation (1a) and 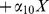 to the 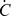 equation (1b) in System (1).
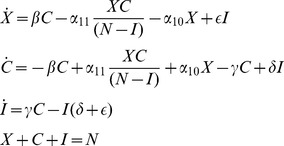
(9)


The reduced system (2) now becomes:
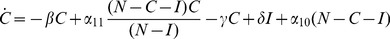
(10a)

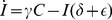
(10b)


There is no longer a crime-free equilibrium, since some proportion of law-abiding citizens will turn to crime on their own. However, we now show that the previous crime-free equilibrium bifurcates to a low-crime equilibrium, and the previous endemic equilibrium bifurcates to a high-crime equilibrium. We will study system (10) by modifying the phase plane analyses that we used in Figures 3, 4, and 5 in our study of system (2).

**Figure 7:**
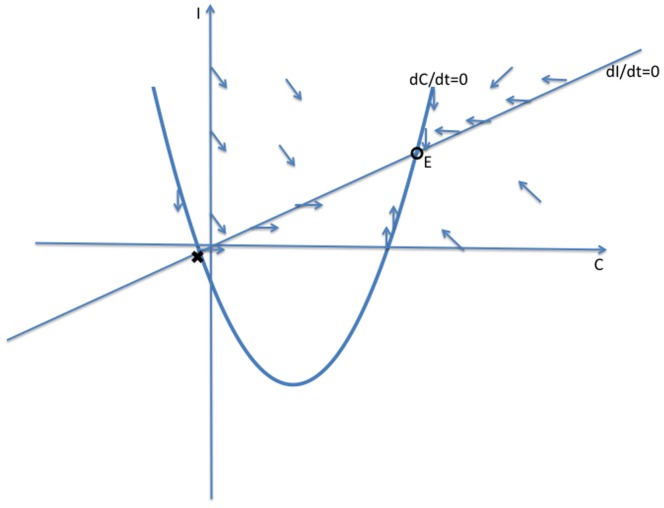
Case 1 for System (10).

**Figure 8:**
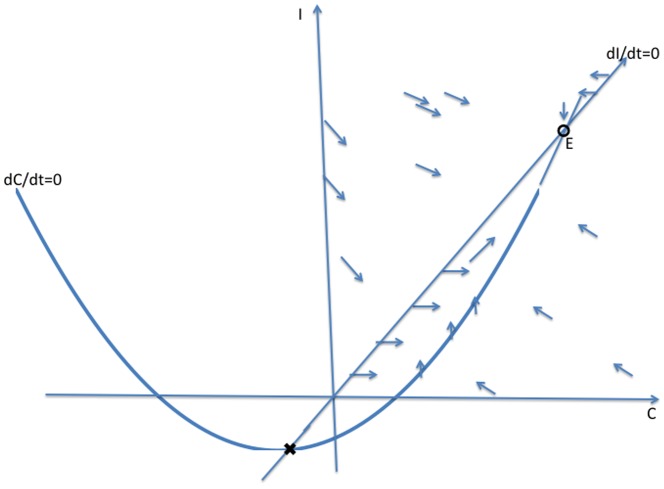
Case 2 for System (10).

**Figure 9:**
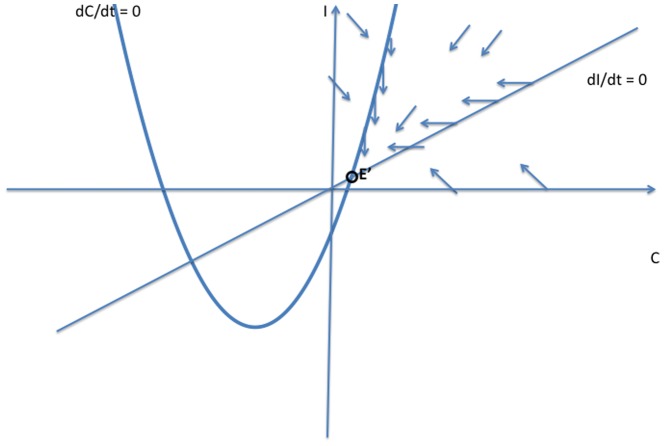
Case 3 for System (10).

In the phase diagrams in Figures 7, 8, and 9, the new 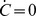 isocline is still a concave-up quadratic curve, but the value of 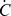 at the origin 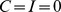 is now 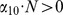, so that the curve 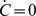 crosses the 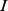-axis below the origin. Figures 7, 8, and 9 show the analogues to Figures 3, 4, and 5 with the new 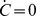 curve below the old one. We see now that the origin 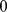 is no longer a steady state, but is replaced in Figures 7 and 8 by a steady state 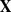 in the negative quadrant. In these two cases, the new (and only positive) steady state 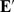 bifurcates from the old endemic steady state 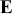 with higher values of 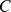 and 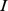, and is globally asymptotically stable. In Figure 9, the crime-free steady state 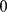 bifurcates to a low-crime steady state, which is globally asymptotically stable.

The threshold condition for the stability of the crime-free equilibrium for Model 1 still holds for the low-crime equilibrium in the bifurcated system. In other words, Figure 9 holds if and only if
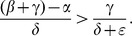



Otherwise, the high-crime equilibrium is the globally stable steady state. Therefore, when some people have a propensity to commit crimes independent of contagion effects, crime will always persist, but there are still two distinctive long-run equilibrium levels of crime. And adding the propensity effect does not change the tipping point that separates convergence to a high-crime equilibrium from convergence to a low-crime equilibrium.

We present a more calculus-based analysis of this system (10) in Section S1 of File S1.

### Model 3: Simplified Five-Dimensional Model

Building on what we learned in the planar system analysis of the 3D models (2) and (10), we begin to move to the Full 5D Model depicted in Figure 1, adding complexities that relate to policy concerns. The first such addition is that of repeat offenders – recidivists. We distinguish those who return to crime after incarceration (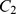) from criminally active persons not yet apprehended (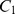). We add two new compartments: 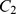 and a transient post-prison compartment 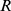 (“recently released”), from which former prisoners can either achieve social mobility and assimilate back into society (rehabilitation) at rate 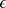 or slip back into crime into the new compartment 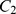 at rates 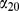 and 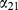. Those in 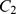 can be reincarcerated (return to 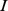) at rate 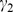 or reform and return to 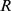 at rate 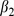. Parameter 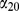 is the propensity for a former criminal to recidivate, independent of influence from others. It can be viewed as the likelihood that time in prison will encourage a return to a life of crime, consistent with the notion of prisons as “schools of crime.” Parameter 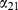 captures the influence of the currently criminally active on the return to criminal activity of the recently released. This flow is given by the following system of equations: (Here and throughout this paper write 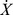 for the time derivative 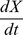.)
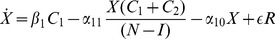
(11a)

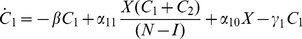
(11b)

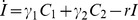
(11c)

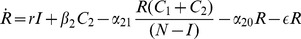
(11d)

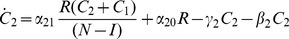
(11e)

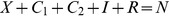
(11f)


Throughout this paper, we refer to system (11) as the “Full 5D Model.” In equation (11a), law-abiding citizens can become criminals either via contact with a criminal or a criminal milieu (“contagion effect”) at rate 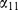 or through a propensity 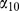 to commit crimes independent of contagion effects. The contagion dynamic is captured by 
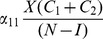
; the non-contagion dynamic by 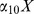 in equations (11a) and (11b). Once citizens turn to crime and become first-time offenders 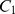, they can reform (move back from 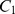 to 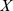) due to non-punitive social interventions (“desistance”) at rate 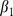, or become incarcerated (move from 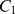 to 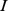) at rate 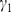 (“incapacitation”). The incarcerated population 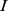 is released from prison (move from 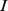 to 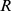) at rate 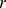, yielding the recently released population 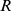 which is targeted by re-entry/rehabilitation programs. Some in the 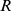 population undergo successful rehabilitation at rate 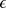 (“redemption”), assimilating back into society as law-abiding citizens 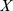. However, others in the 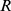 population become repeat-criminals and enter 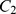 (“recidivism”). This dynamic may occur via interactions with criminals on the street, captured by the 
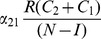
 term, or by a propensity to recidivate after prison, captured by the 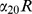 term in equations (11d) and (11e). (The 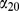 dynamic is related to the role that prisons play as “schools of crime.”) Recidivists 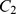 will either be incarcerated (return to 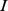) by the 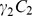 term or reform (return to 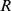) by the desistance term 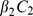 in (11e). The last equation (11f) is simply a reminder that total population size is conserved, so that we can eliminate one of the equations in our analysis.

In our first analysis of “Full 5D Model” (11), we assume: 1) that all first time offenders turn to crime because of the influences of current criminal activity, so that 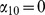, and 2) that no contagion effect is needed to return to crime after a prison term, so that 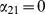. We use this 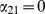 assumption only to simplify the calculation of the endemic equilibrium; we will remove it when we calculate the expression for the threshold 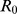. We use the contagion-only assumption 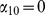 exactly for the reasons we did in working with the 3D Models 1 and 2: we need the existence of a crime-free equilibrium because the Lyapunov function approach works most easily when one wants to derive conditions for all orbits to go to the *origin*. The compartmental diagram for this simplified version of system (11) is that of Figure 1 with 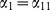 and 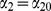.

#### The Endemic Equilibrium

We first calculate the endemic equilibrium for system (11) with 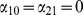. In this case, at equilibrium, the four equations (11b) to (11e) yield:
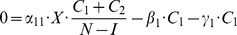


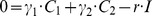


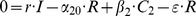


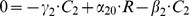
Rewrite these equations as:
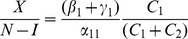
(12a)

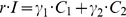
(12b)

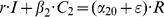
(12c)

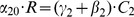
(12d)Combine the last three equations as:
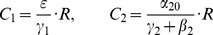
(13)Plug these into (12a) to obtain:
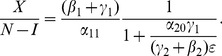
(14)In the next Subsection, we will see that the basic reproduction ratio for this system is
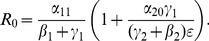
(15)So, once again, at the endemic equilibrium:
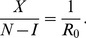
(16)


We will interpret expression (14) in the next subsection after we compute its 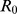 and we will see what it implies for various interventions in Section entitled “Analysis of Policy-Related Changes in the 5D Model.”

#### Threshold for the Stability of the Crime-Free Equilibrium in Model 3

As we did for the simple 3-dimensional system (1), we use a Lyapunov Function approach to derive the threshold for the global asymptotic stability of the crime-free equilibrium of the simplified system (11). We will even re-include a contagion term for the recidivism transition from 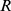 to 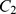. In other words we will work with our Full 5D Model (11) with 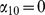, a restriction we will remove below. Since 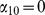, the crime-free equilibrium is still, of course,
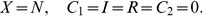
(17)


We carry out the details of the Lyapunov function approach In the following subsection. For those who wish to avoid the mathematical details of that computation, we present the bottom line here: the threshold condition for the (global) stability of the crime-free equilibrium is
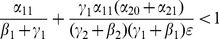
(18)or, equivalently,
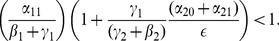
(19)


The two (equivalent) expressions on the left sides of (18) and (19) can be thought of as *basic reproduction ratios* for the spread of crime.
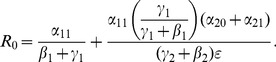
(20)In the first term in expression (20), the numerator is the rate of movement from 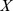 to 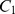 and the denominator is the sum of the two rates for leaving 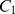, desistance 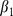 and incarceration 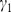. In the second term in (20), the numerator tracks the rates of movement from 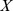 to 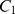 (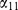), the movement from 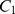 to prison 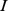 of those that didn't desist back to 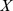 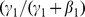), and the movement from prison to 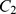 (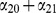). The denominator includes the three different ways to move out of 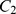: via desistance 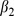, re-incarceration 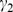, and redemption 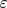. So the first term in 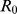 is the input/output ratio of movement from 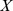 to 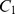, the second term is the input/output ratio for movement from 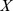 to 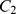. In the Section “Analysis of Policy-Related Changes in the 5D Model,” we will examine expression (20) more carefully to see what it says about the various intervention possibilities.

Note that the 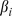
s enter only in the sums 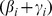 and that 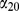 and 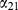 appear only as the sum 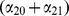 even though that 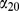 and 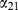 enter differently into system (11).

**Remark:** Of course, if we set 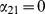 in (20), we retrieve the results of Subsection “Endemic Equilibrium” for System (10), and in particular that
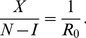
(21)


However, simulations show, that (21) with 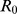 given by (20), does not hold for the expanded system (11) with 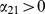, as it does for system (10).

#### Derivation of the Threshold for the Stability of the Crime-free Equilibrium for the 5D Model

Here we carry out the Lyapunov function approach to derive conditions for the crime-free equilibrium (17) to be an asymptotically stable equilibrium for system (11), working with the last four equations of system (11) with 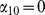, namely, (11b), (11c), (11d), and (11e), with 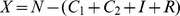 in equation (11b). Once again we look for a Lyapunov function of the form
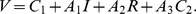
We seek constants 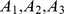, all positive, such that the derivative 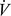 of 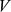 along solutions of (11) is negative. In this case, all solutions tend to the origin.
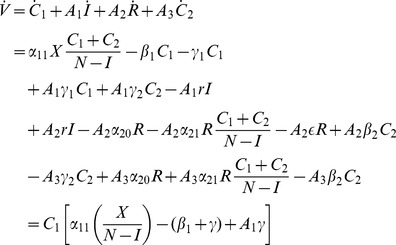
(22)

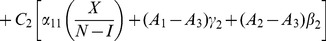
(23)

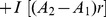
(24)

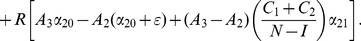
(25)


We seek positive values of 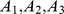 so that the coefficients in the square brackets in (22), (23), (24), and (25) are negative.

For (22), this means:
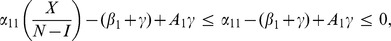
requiring
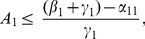
(26)and in particular, that
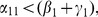
(27)We have seen this condition in even the simplest models.

For (24), to be negative:
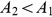
(28)For (23) to be negative:
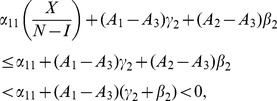
(29)using (28). Write (29) as:
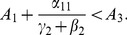
(30)For (25) to be negative:
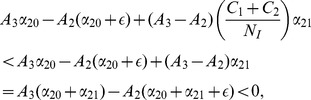
which we write as:
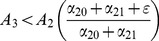
(31)Combining (28), (30), and (31):
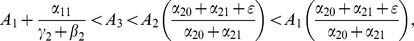
or
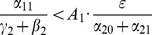
(32)or
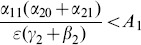
(33)Combining (26) and (33), we write our condition as:
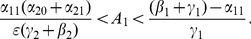
(34)


There exists an 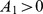 to satisfy (34) (and the appropriate positive 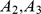), if and only if:
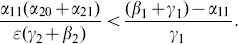
(35)


So, (35) is our threshold condition for the (global) stability of the crime-free equilibrium! Rewrite (35) as:
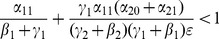
(36)or, equivalently,
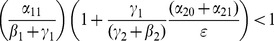
(37)


### Model 4: Beyond Contagion, More Complex 5-Dimensional Model

We now include the possibility that citizens can turn to crime on their own, without the influence of crime around them, as in Section entitled “Model 2: Beyond Contagion, a More Complex 3-Dimensional Model” for the simpler 3-D model. The result is the Full 5D Model (11). The equilibria are the solutions of the following system:
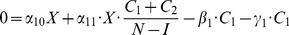
(38a)

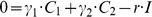
(38b)

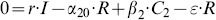
(38c)

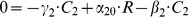
(38d)Rewrite these equations as:
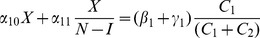
(39a)

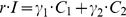
(39b)

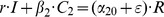
(39c)

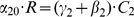
(39d)


As we did in (13), we solve the last three equations as:
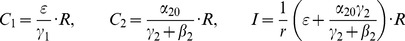
(40)


We can now apply the generalizations of the arguments for Model 2. The analogue of the geometric argument of Subsection entitled “Model 2: Beyond Contagion, a More Complex 3-Dimensional Model” takes note of the fact that the solutions of System (39) in the 4-D 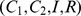-space occur at the intersection of the line given by (40) and the quadratic 3-D surface (“manifold”) given by (39a). That surface moves down, relative to the 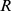-axis, as 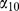 increases from 0, while the line (40) remains unchanged, analogous to the changes in going from Figures 3, 4, and 5 to Figures 7, 8, and 9. If the line crosses the surface only at the origin in the non-negative orthant, then the new crossing with 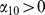 occurs in the strictly positive orthant (as in Figure 9) and the crime-free equilibrium bifurcates to a low-crime equilibrium. Otherwise, the lowering of the surface given by (39a) leads to the line and surface crossing “below” the origin in the negative orthant, so that the only non-negative equilibrium bifurcates “up” from the endemic equilibrium of the 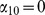 case.

We discuss these results further in Section S2 of File S1.

### Analysis of Policy-Related Changes in the 5D Model

#### Changing the Enforcement/Incarceration Parameter

Expressions (19) and (20) tell us that in order to push a society towards a crime-free equilibrium, we need the contagion/transmission factors, given by the 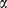
's (in the numerators) small and the retreat from crime rates given by the 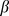
's and 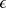 large (in the denominators), as well as the enforcement factors given by the 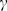
's. Of special interest is the fact that one enforcement parameter 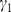 appears in the numerator of (19) and (20) — suggesting that the over-incarceration of first-time offenders can be counter-productive. However, it is balanced by a 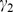-term in the denominator in formulation (19), suggesting that there should be more attention paid to apprehending repeat offenders (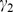) than first time offenders (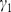). The 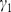 in the numerator in (19) is multiplied by
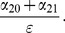
(41)


We can work with expression (19) to gain insights on various interventions. For example, we can compute how 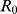 changes as the incarceration parameter 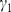 increases:
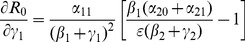
(42)


When (41) is small, in particular, when
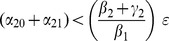
(43)so that recidivism 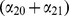 is small relative to rehabilitation and redemption, then increasing 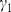 does decrease long-run crime prevalence. However, if recidivism becomes larger relative to successful rehabilitation so that (41) increases, then once (43) is violated, increasing enforcement on first-time offenders 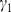 leads to higher crime prevalence. *If people who are released from prison are likely to return to crime and unlikely to assimilate successfully back into society, then increasing the incarceration rate for first time offenders will have negative long run consequences.*

Alternatively, in formulation (20) the 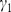 is divided by a 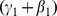 term. In the latter case, the 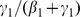 factor is small provided 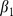 is larger than 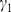, emphasizing the importance of social interventions.

We do see from expression (20) that increasing enforcement 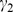 on recidivists unambiguously leads to lower crime prevalence.

#### Effect of Prison Term Length

**Figure 10:**
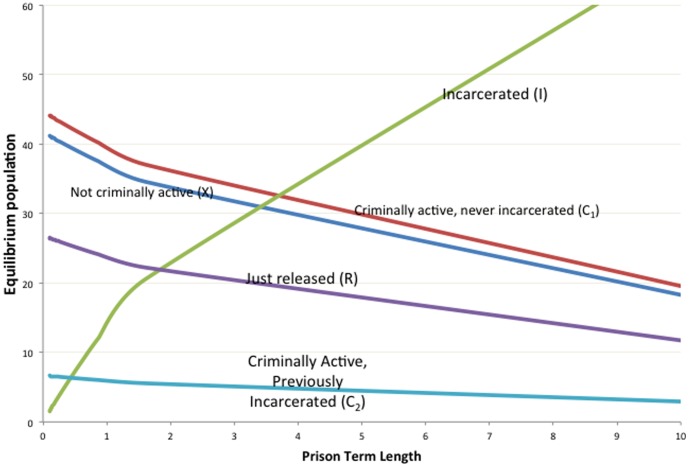
Effect of Prison Term Length.

It is especially interesting to note that the crime-free threshold 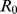 and the equilibrium “prevalences”, that is, the fraction of the non-incarcerated population that is either is criminally-active 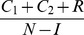 or not criminally-active 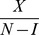, of the last two subsections are independent of the rate of prison release 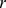 and therefore of the length of prison term 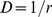. However, as equations (12b) and (12c) indicate, the equilibrium *values* of the individual variables 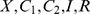 do depend on 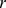. Figure 10 provides an example of this dependence for a somewhat arbitrary choice of the parameters (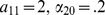, 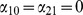, 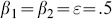, 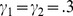). In Figure 10, we see that as the term length 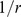 increases (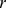 decreases from 10 to .1), the equilibrium 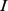 increases, while the other variables decrease. We now show that this is true in general.

Equation (16) tells us that 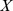 and 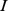 move in opposite directions as 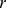 increases. Equation (13) tells us that 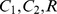 move in the same direction as 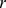 increases, and that we can write 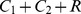 as a positive multiple 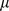 of 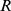. Write (16) as
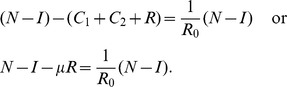
Rewrite this as
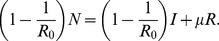



Since 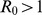 and both coefficients on the right side are positive constants as 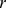 increases, it follows that 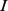 and 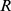 move in opposite directions as 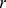 increases. Combining this with (12b) implies that as 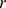 increases, 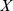, 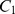, 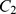, 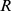 increase and 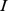 decreases, as illustrated in Figure 10.

We conclude that increasing the prison term length has no effect on the long-term fractions of the population that is criminally-active and on the crime-free threshold, but it does lead in the long run to a larger prison population, fewer criminally active, and fewer non-criminally active individuals.

Also, as Figure 10 suggests, there are diminishing returns to longer prison lengths. With the average cost of a prisoner at over $35,000 a year, one can use Figure 10 to take into consideration the societal costs and benefits of longer prison terms and choose accordingly.

#### Effect of Long-term sentences for the 5D Model: Eliminating Parole

**Figure 11:**
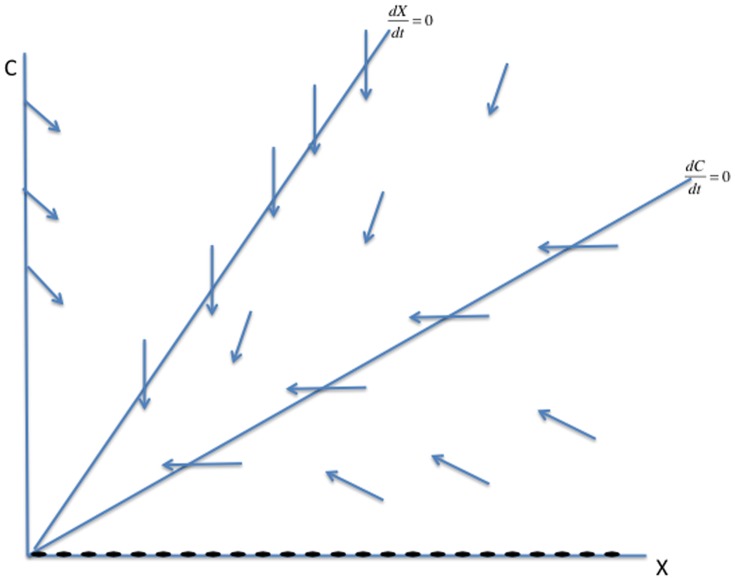
Phase Portrait of System (44) for *R*_0_>1.

**Figure 12:**
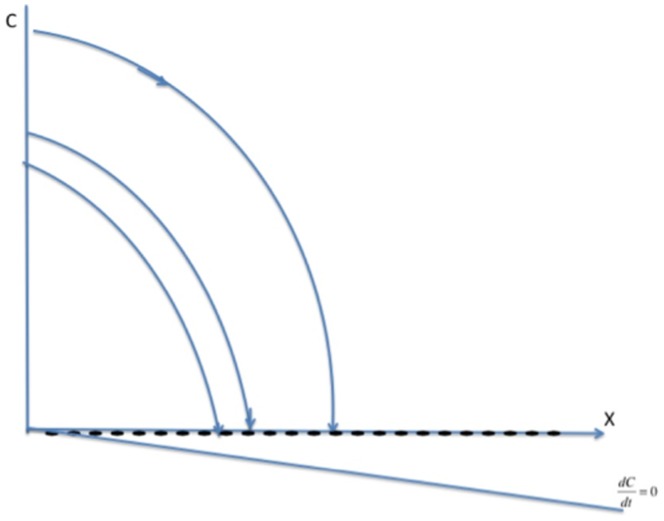
Phase Portrait of System (44) for *R*_0_<1.

It is interesting to consider how slowing the rate of release (tightening the spigot) affects the crime rate, i.e., what happens as 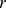 gets small (and prison term length get large). In this vein, we examine the limiting case in which no one is ever released from prison. Since there is no release from prison, there is no parole 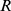 and no repeat crime 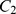. Model (45) simply becomes:
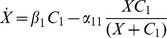
(44a)

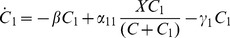
(44b)

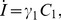
(44c)a 3D system that we threat as a 2D system in 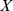 and 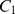. The crime-free threshold for this system is simply 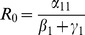. In Figures 11 and 12 we draw the two phase diagrams for this system, depending on whether 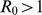 (Figure 11) or 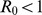 (Figure 12), in words, whether the “reproduction” of criminals is above or below the “replacement”value. As shown in Figure 11, when 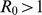, all orbits tend to the 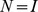 (everyone in prison) equilibrium at the origin (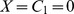). It's not that crime has been eradicated, but that the entire non-incarcerated population has been eliminated.

On the other hand, if 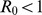, the system moves toward a crime-free equilibrium as the segment of the population in 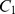 (criminally-active but not incarcerated) is diminished either because they become incarcerated (move to 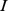) or desist from crime (move to 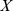). However, there are multiple crime-free equilibria when 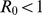, with different fractions of people in 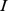 (incarcerated) and 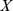 (not incarcerated but also not criminally active), depending on the initial level of criminal activity in the population. In this no-parole environment, even if 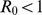, i.e., the lures into crime are smaller than the lures to reform, some people will get trapped into the prison system.

**Figure 13:**
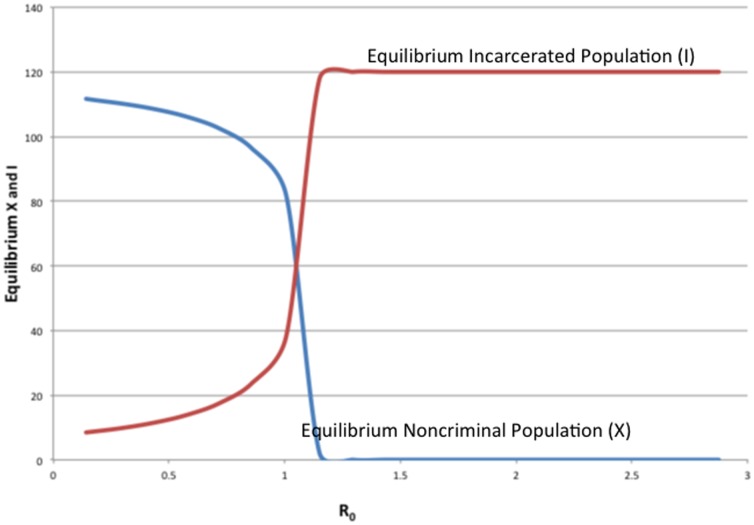
Final *X* and *I* as *R*_0_ changes.

Figure 13 shows the dramatic changes in the long run crime-free and prison populations as 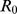 crosses the threshold 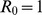. In the simulations behind this Figure, we vary only 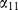, holding the other parameters at fixed values, including 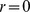. 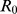 is a linear multiple of 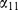 with 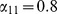 corresponding to 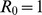. When 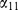 is 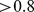, 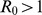 and the entire (120-member) population is incarcerated. When 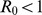 and decreases even further, everyone in the community is either not criminally active or incarcerated, with the equilibrium number of incarcerated decreasing as 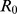 decreases. When 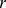 is small but positive, the corresponding graphs are similar to those of Figure 13.

There is Lyapunov function for system (44): 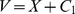. In this case, 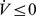 independent of the value of the tipping point 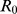; 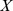 and 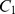 decrease over time and the prison population grows. If 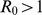, the prison population eventually includes everyone. If 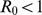, the criminally active who did not desist will be incarcerated forever.

The situation is even more dramatic if we eliminate parole in system (39) in which some turn to crime independent of the level of criminal activity around them. In this case, we include 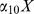 terms in equations (44a) and (44b). The same Lyapunov function 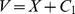 works for this case as it did in the contagion-only model just above. In this case, however, the only equilibrium for the modified system (44) is 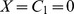, 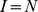, and all orbits of the modified (44) tend to it, no matter whether 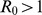 or 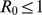. Everyone is eventually incarcerated.

#### Effects of Desistance Parameters

It is worth considering the effects of the parameters 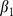 and 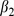, reflecting the rates at which first-time criminals and recidivists discontinue criminal activity, respectively. One easily computes that partial deriviatives: 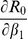 and 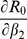 are both negative, and therefore social interventions targeting the desistance of active criminals unambiguously decreases crime.

For most choices of reasonable parameters, our simulations have found that 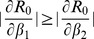. (For example, this inequality holds when 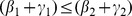.) In those cases, social interventions targeting the desistance of criminals who have not yet been incarcerated plays a larger role in driving communities towards a low crime equilibrium than those targeting the desistance of recidivists. In some sense, this is consistent with Frederick Douglas's assertion that “It is easier to build strong children than to repair broken men.”

### Model 5: Modeling a Three-Strike Policy with Leakage

As an application of the abstract approach of this paper, we examine a concrete policy intervention for discouraging repeat crime, namely the “three strike policy,” wherein a criminal convicted a third time faces a mandatory life sentence. We examined a simplified version of this in the previous subsection, namely mandatory life sentences for any offense. To treat the three-strike policy, we need to expand our model to distinguish first time, second time and third time arrested offenders.

**Figure 14:**
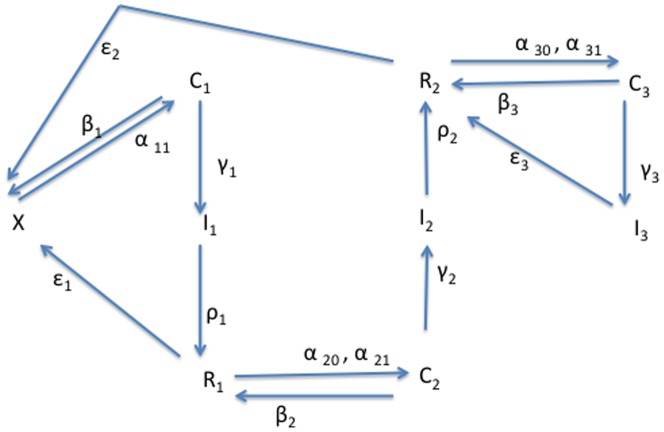
Flow Diagram for System (45).

As we did in the previous section, we begin by allowing some leakage from the mandatory life sentence (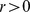), before we examine the stricter regime (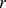 equal or nearly equal zero). In fact, a life sentence after a third strike is often not a “true” life sentence (without any chance of release). In the real world, 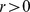 among the lifers. Figure 14 presents the flow diagram for this system. Section S3 in File S1 provides a summary list of the variables and parameters used in model (45). Since we now have possibly different prison term lengths for first-, second-, and third-time incarcerations, we use 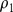, 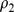, and 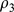 to represent these three rates of incarceration.
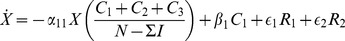
(45a)

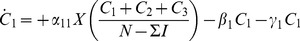
(45b)

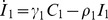
(45c)

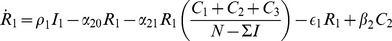
(45d)

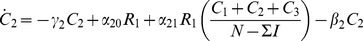
(45e)

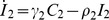
(45f)

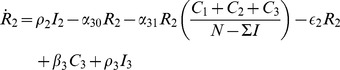
(45g)

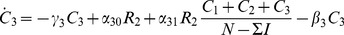
(45h)

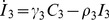
(45i)where
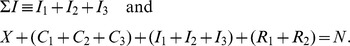



#### Stability of the Crime-Free Equilibrium for Model (45)

The crime-free equilibrium has
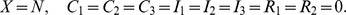



We use the same Lyapunov function approach to find its stability conditions, using the last eight equations. The Lyapunov function derivation of the threshold is carried out in Section S4 of File S1, where we compute that the threshold for global stability of the crime-free equilibrium is:
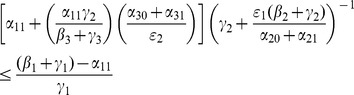
or
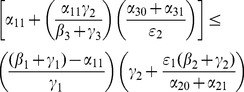
(46)


With a bit of algebraic manipulation, rewrite (75) in each of the following three ways:
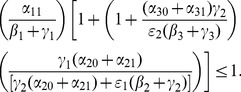
(47)

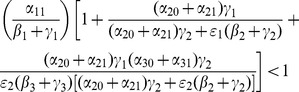
(48)

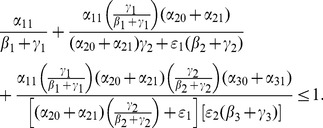
(49)


Expression (49) for the 9D system (45) has strong similarities to the corresponding 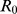 expressions (19) and (20) for the 5D system (11) (with 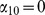). The three numerators in (49) are the input paths from 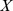 to 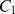, 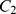 and 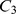 respectively. The three denominators in (49) are a bit more subtle, but they strongly relate to paths out of 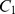, 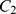, and 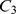 respectively. Note once again that the 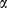
s appear only in pairs as the “onset” expressions for the 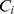
s. Each 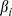 appears only with the corresponding 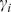 as the combined social program/enforcement parameter 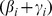 for the removal of active criminals from the population.

#### Endemic Equilibrium for System (45)

As we did for the simpler three dimensional Model 1, we will compute the endemic equilibrium for system (45), with the simplifying assumption that there is no contagion factor for repeat criminals. We will show that this equilibrium has the form 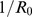 for the 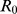 on the left hand side of (48). So we return to system (45), but with 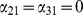.

First, set the left hand sides of each equation in (45) equal to 0. Combine the new (45i), the new (45g), and (45h) and (45f) to compute that
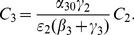
(50)Combine (45c), (45d), and (45e) to compute:
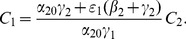
(51)Finally, write (45b) as:
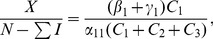
(52)Substitute for 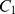 and 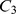 into this equation from (50) and (51). After a bit of algebra and cancellation of the 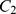
s, one finds:
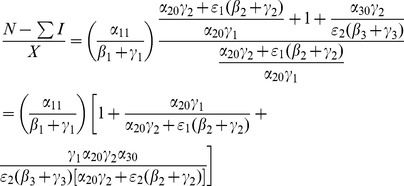
(53)Comparing this to the 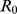 formula (48) with 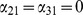, we find again that indeed
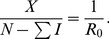
(54)


#### Effect of Prison Term Length in Model (45)

Once again, as we see in (53), the equilibrium fraction of the population that is not criminally-active
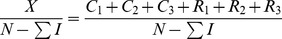
(55)and the crime-free threshold 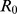 as in (49) of the last two subsections are independent of the rate of prison release 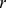 and therefore of the length of prison term 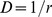. However, as equations (45g) and (45i) set equal to zero indicate, the equilibrium values of the individual variables 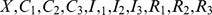 do depend on values of the 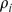
s. In fact, an analysis vey similar to that in the Section on “Effect of Prison Term Limits” still holds. Just as in Figure 10, as the term lengths increase (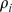 decreases), the equilibrium 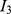 increases, while *all* the other variables decrease, including 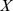 and the 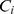
s. Increasing the prison term length has no effect on the long-term prevalences and the crime-free threshold, but it does lead to more people in prison, fewer criminals and fewer crime-free individuals in the long run.

### Model 6: 9-Dimensional System With a Three Strike Policy

**Figure 15:**
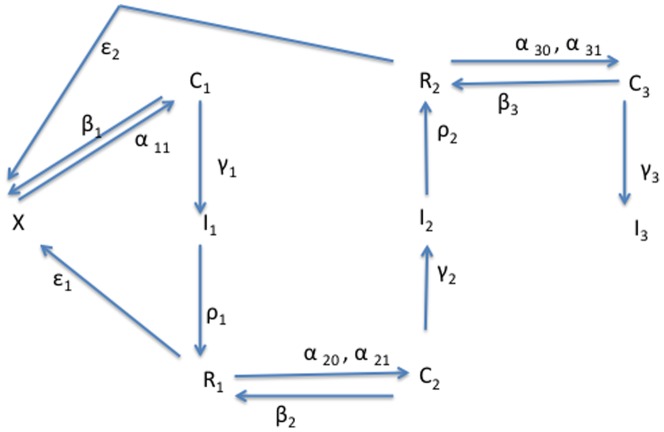
Flow Diagram for Three-Strike Policy.

To model the Three-Strikes-And-You're-Out Model — mandatory life sentences after a third conviction, we set 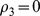 in system (45). Figure 15 presents the corresponding flow diagram. Just as we saw for the 5D Model and illustrated in Figure 11, when 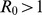, all orbits tend to the everyone-in-prison equilibrium at the origin (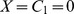). On the other hand, if 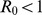, the outcome depends somewhat on the initial conditions. There are no criminals in the long run (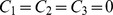), those who became criminals for a while either reform via 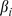 and end up in 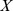, or they are arrested the third time and spend the rest of their lives in prison. Proofs can be found in Section S5 of File S1.

## Results and Discussion

The spread of crime is a complex problem requiring systems-level thinking. Effective intervention requires more than optimally allocated police forces. In this section, we summarize our models, compare the expressions we computed for the tipping points and endemic equilibria, and summarize the policy implications we derived from those expressions.

### Summary of Model Analyses

We have worked with a number of parameters which can influence crime outcomes, including onset of crime, incarceration, recidivism, desistance, and redemption.

- the rate at which individuals turn to crime the first time, either by themselves (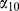; propensity to crime) or through interactions with the crime and the criminally active around them (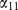; contagion effect of crime),
- the rate at which the criminal-justice system imprisons those with and without criminal records (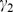 and 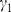 respectively; incarceration),
- the (recidivism) rate at which previously incarcerated criminals return to crime, either by themselves (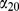; propensity to recidivate) or through interactions with the crime and the criminally active around them (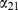; contagion effect of crime on former criminals).
- the success rate of non-punitive measures in reforming criminals with and without criminal records (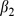 and 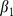 respectively; desistance),
- the rate at which those with criminal records not only desist from crime but also achieve “redemption,” meaning that their criminal record is, for all intents and purposes, no longer consequential (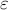; rehabilitation/redemption),

As stated in the Introduction, the purpose of working with abstract, data-free models as a precursor to empirically-informed work is to understand the relationships among these parameters in the spread of crime.

The focal model of this paper is Model 4, the Full 5D Model given by system (11), which includes recidivism and prison term length. To develop intuition for this model, we also worked with a bare bones 3D Model (Model 1, system (1)). To test the water for more complex models and interventions, such as the three-strike policy, we analyzed a 9D model (Model 5, system (45)), which kept closer track of the level of recidivism. For all these models, under the condition 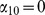 (all crime initiated through contagion), we computed an analytic expression for the tipping point between the crime-free equilibrium and the high crime (endemic) equilibrium in terms of the model's parameters. We now summarize the main results from our analysis.

- Our computation showed that there is exists a **unique high-crime equilibrium** in each model.
- The necessary and sufficient conditions for **global convergence to the crime-free equilibrium** in the 3D, 5D, and 9D models, respectively, are:
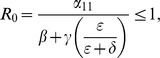


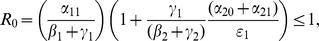


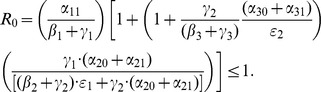
 One can begin to see patterns emerge in these expressions as we increase the model's complexity.
- To further the motivation for the above three expressions for tipping points, we write them as **input-output** ratios:
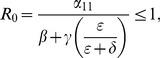
(56)

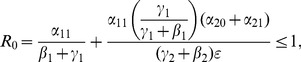
(57)

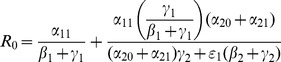
(58)

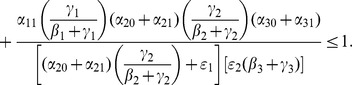

We see that the two numerators in (57) describe movement from 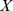 to 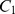 and 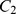 respectively, while the two denominators give the ways of moving out of 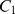 and 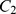 respectively. The first term in (57) is the input-output ratio for 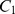, the second term is the input-output ratio for 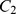. The 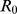 in (56) is truly a “reproduction number”: the number of non-criminally active that a criminally active person can seduce into crime while he or she is not incarcerated.

- When we relax the assumption that people turn to crime only through contagion (so that 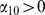, as in Models 2 and 4), a crime-free society is no longer possible. **However, the same tipping point inequalities determine whether a society converges to a low crime or high crime equilibrium**.
- Despite the fact that they enter the systems in different ways, the parameters 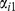 and 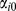 that model the transition into criminal activity, either with or without the influence of other criminals, respectively, always appear together as a sum, capturing the dynamic known as “**onset**.”
- Similarly, the parameter 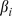 that represents inducements for the desistance of criminal activities only appears in the above expressions as a summand with the corresponding incarceration parameter 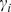, possibly representing the **role of general deterrence** in moving away from criminal activity.
- **Relationship between** 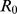 **and endemic prevalences:** In Models 1, 3, and 5, when the above 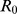
s are 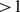, the crime-free equilibrium is unstable and all orbits converge to a unique “endemic” equilibrium. At this high-crime equilibrium, the fraction of the non-incarcerated population that is not criminally-active 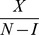 is given by 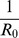 and the fraction that is criminally-active is given by
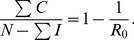
(59)

### Summary of Policy Implications

It follows from (59), parameter changes that increase 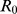 also increase the fraction of the population that is criminally-active. Therefore, we worked with expressions (56), (57), and (58) to evaluate the relative effectiveness of various intervention strategies.

- We can use our analyses to determine the necessary level of incarceration to reduce long-run crime under Model 1's assumptions, writing (56) as
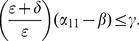
(60) Under Model 1's assumptions, if social programs are even slightly more effective than the transmission rate of criminal behavior — that is, if 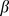 is larger than 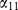 — no incarceration is necessary for crime to continually decrease to a low equilibrium. If 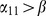, however, inequality (60) gives the minimal amount of incarceration needed to reduce crime to a low equilibrium.
- To consider the effect of increasing incarceration on first-time criminally-active offenders, we differentiate 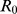 with respect to 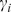 to take advantage of the expressions we have for 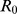. For example, focusing on (57), we compute
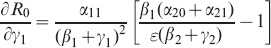
(61)When recidivism 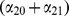 is small relative to rehabilitation and redemption, then increasing 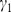 does decrease long-run crime prevalence. However, if recidivism is more likely than successful rehabilitation, increasing incarceration on first-time offenders 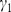 leads to higher crime prevalence (and **decreasing incarceration leads to lower crime prevalence**). Intuitively, if the “failure” rate among people coming out of prison is too high, then incarcerating so many people is counterproductive. There are social and economic benefits to successfully helping returning prisoners reintegrate.
- On the other hand, the corresponding 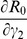 is always negative for Model 3. Increasing enforcement/incarceration against recidivists does reduce the prevalence of crime.
- Similarly (and obviously), 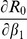 and 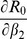 are always negative.Increasing desistance also reduces the prevalence of crime.
- In the above expressions for 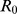, the 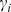 is always divided by a 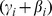 term. This ratio 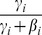 is more sensitive to increases in desistance incentives 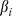 than to increases in incarceration 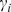. In other words, **in driving a community to a low-crime equilibrium, successfully increasing the rate of desistance (through social interventions, for example) is systematically more effective than increasing the rate of incarceration**.
- We also see that 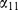 is the only parameter that appears as a multiplier for the entire expression of 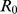, emphasizing that **the long-run equilibrium level of crime and incarceration is extremely sensitive to changes in the likelihood that an individual turns to crime in the first place, especially onset that is influenced by social interactions with other criminally active people**. This is not unlike the medical saying that “prevention is the best cure.” Accordingly, finding ways to decrease 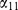 should be a major priority. For example, if children who have poor access to education are far more likely to turn to crime than their counterparts, then improving educational access will systematically decrease long-run crime and incarceration levels, which has both social and economic benefits.
- The 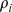
s and 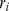
s, which are key indicators of sentencing length, do not appear in the expressions for either the tipping point or the prevalence of crime. At least in these simple models with long-term foci (such as tipping points and endemic equilibria), **the reduction and endemic prevalence levels of crime are independent of the length of sentence.**
- But while increasing the prison term length has no effect on the long-term prevalences and the crime-free threshold, it does lead to **more prisoners, fewer criminals, and interestingly fewer law-abiding citizens in the long run**, assuming a fixed population 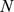.
- **There is also a decreasing returns effect for prison term lengths**, that is, increasing prison term lengths have smaller and smaller overall effects on the amount of crime, as Figure 10 suggests. **In particular, longer sentences can lead to greater increases in the prison population than decreases in the criminal population**.
- If 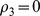 (mandatory life sentences for the third conviction), there are no criminals in the long run (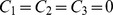). If 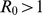, and everyone eventually spends their lives in prison. If 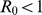, those who became criminals for a while either reform via 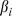 and end up in 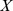, or they are arrested the third time and spend the rest of their lives in prison. If, independent of a contagion effect, people have even the smallest propensity towards criminal behavior (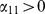), then everyone eventually spends life in prison independent of the size of 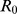.

## Conclusions

In this paper we have presented a series of increasingly complex mathematical models of the spread of crime in a population and studied the relationships among the models' parameters. Although these abstract models do not generate empirical findings, they lay a foundation for future empirical work on the macro-level dynamics of crime and punishment, thus rejuvenating a rich but somewhat dormant tradition of systems models of crime. The main contributions of our current work are threefold. First, we showed that each of the systems we analyzed had both a low and high-crime equilibrium, and we demonstrated how to derive analytically the threshold 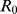 that defines the tipping point between the low- and high-crime equilibria. We also demonstrate how the threshold can be viewed as the sum of input-output ratios comparing the movement of people into the crime (via initial onset and recidivism) to the movement of people out of crime (via incarceration, desistance, and redemption). These threshold expressions also reveal that the relative size of the criminally-active population is more sensitive to changes in the rate of desistance away from crime than it is to incarceration, suggesting that policy efforts aimed at encouraging desistance (e.g., prisoner reentry programs and social programs targeting young criminals and juvenile delinquents) may be a more efficient way to reduce crime compared to increasing incarceration rates. The threshold expressions also indicate that policies and interventions that reduce the likelihood of being lured into crime in the first place are of the utmost importance in reducing the long-run levels of crime.

Another contribution of our work is to specify the conditions under which increasing the punitiveness of criminal sanctions – albeit in a way that does not distinguish between higher levels of arrest, conviction, or sentencing to incarceration – will lower the crime rate. Differentiating 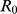 with respect to 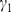 (the rate at which people are incarcerated for the first time) revealed that increasing the incarceration rate of first-time offenders will only diminish the crime rate if the forces propelling people into crime, 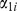 (onset) and 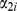 (recidivism), are small relative to the forces driving people away from crime, 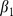 and 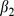 (desistance) and 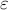 (rehabilitation/redemption). In a world where the recidivism rate outpaces the rehabilitation rate among formerly incarcerated people, increasing the rate at which first-time offenders are incarcerated becomes counterproductive, driving the system toward a higher crime rate. However, increasing the rate of incarceration among recidivists will reduce the long-run level of crime in the system.

A third contribution of the models presented in this paper is that they shed light on the system-level consequences of increasing the length of prison terms. The long-run effect of increasing the length of prison terms are to increase the fraction of the population that is incarcerated, but it has no effect on changing the fraction of criminally-active people in the population. Moreover, as we showed in Figure 9, there are diminishing returns to increasing the length of prison sentences.

Abstract models like those presented in this paper, if accepted as reasonable approximations, can be used to calculate hard-to-estimate parameters, such as the contagion effects, as has been done for models of the spread of disease [48]. In other words, if there is agreement on the long-term crime level in an area, and on the values of many of the underlying parameters (such as the incarceration probability), the models' equations can be used to estimate other parameters. Because our conclusions follow from careful mathematical analyses, controversial or incorrect model conclusions lead to challenges to the models' assumptions — a valuable exercise in itself.

We recognize the limitations of these models in their current form, largely due to the simplifying assumptions, including spatial homogeneity, fixed transition probabilities between populations, and a fixed population. The ultimate benefit of the modeling approach begun in this paper lies in future work, in which we intend to relax the models' restricting assumptions, account for population heterogeneity (e.g., by age, race, and socioeconomic status), allow for variation by type of crime, and incorporate more stages of the criminal justice system into the models.

## Supporting Information

File S1 **Supporting information and figures.** In File S1, we carry out in some detail the calculus and algebra calculations needed to compute endemic equilibria, compute formulas for tipping points, and analyze extensions of simpler models presented in the body of this paper.
(PDF)
