# Supplementary material for: Modeling the Underlying Dynamics of the Spread of Crime
Source: PLoS One. 2014 Apr 2;9(4):e88923. doi: 10.1371/journal.pone.0088923 (PMC3973672; doi:10.1371/journal.pone.0088923)
Supplement: File S1 — Supporting information and figures. In File S1, we carry out in some detail the calculus and algebra calculations needed to compute endemic equilibria, compute formulas for tipping points, and analyze extensions of simpler models presented in the body of this paper. (PDF) [file pone.0088923.s001.pdf]

File S1. Supporting Information

S1. Model 2: Beyond Contagion (Analytic Approach)

In this section we give a calculus-based analogue to the geometric arguments in the Section entitled "Model 2: Beyond Contagion, a More Complex 3-Dimensional Model" for the 3D system (10) with $\alpha_{10} \neq 0$. This calculus-based approach is important in our examination of higher-dimensional cases. Substitute

$$I = \left(\frac{\gamma}{\delta + \varepsilon}\right) C \tag{S1}$$

from the right hand side of (10b) into the right hand side of (10a) to obtain a family of curves

$$G(C, \alpha_{10}) \equiv -(\beta + \gamma)C + \alpha_{11} C \frac{\left(N - \left(\frac{\gamma + \delta + \varepsilon}{\delta + \varepsilon}\right) C\right)}{\left(N - \left(\frac{\gamma}{\delta + \varepsilon}\right) C\right)} + \delta \left(\frac{\gamma}{\delta + \varepsilon}\right) C + \alpha_{10} \left(N - \left(\frac{\gamma + \delta + \varepsilon}{\delta + \varepsilon}\right) C\right).$$

parameterized by α_{10}. For each α_{10}, the zeroes of $C \to G(C, \alpha_{10})$, along with (S1), give the components of the zeroes of system (10a), (10b).

When $\alpha_{10} = 0$, $G(C, 0) = 0$ has two solutions $C = 0$ and another one $C = C^*$, which can be computed from (7). For $\alpha_{10} = 0$, we examine two cases, using the results of Subsection "Phase Plane Analysis of System (2)."

1. If $C^* > 0$, then C^* is the C-component of the (globally stable) endemic equilibrium of the system.
2. If $C^* < 0$, then $C = I = 0$ is the unique equilibrium of the system, and it is globally stable.

Inequality (4) separates these two cases.

Write $C(\alpha_{10})$ for the zero of $G(\cdot, \alpha_{10})$ that bifurcates from $C = 0$ as α_{10} increases from 0, i.e.,

$$G(C(\alpha_{10}), \alpha_{10}) = 0, \quad \text{and} \quad C(0) = 0. \tag{S2}$$

Use the Implicit Function Theorem to compute how C changes with α_{10}:

$$\begin{aligned} \frac{dC}{d\alpha_{10}}(0) &= -\frac{\frac{\partial G}{\partial \alpha_{10}}(C(0), 0)}{\frac{\partial G}{\partial C}(C(0), 0)} \\ &= -\frac{N}{\alpha_{11} - \left(\beta + \frac{\varepsilon\gamma}{\delta + \varepsilon}\right)}. \end{aligned} \tag{S3}$$

From the Subsection on "Phase Plane Analysis of System (2)," Case 1 holds when the denominator of (S3) is positive and so $\frac{dC}{d\alpha_{10}}(0) < 0$. Therefore, $C(\alpha_{10})$ is negative for (small) positive α_{10}. This means that there is now only one non-negative root, and it's the one that bifurcates from the endemic equilibrium. Case 2 above holds when the denominator of (S3) is negative, and so $\frac{dC}{d\alpha_{10}}(0) > 0$, i.e., C is increasing in α_{10}. In this case, the root $C = 0$ bifurcates to a strictly positive root, and the crime-free equilibrium bifurcates to a low-crime equilibrium, while the other root of G remains negative.

Incidentally, let

$$H(C; \alpha_{10}) \equiv G(C, \alpha_{10}) \cdot \left(N - \left(\frac{\gamma}{\delta + \varepsilon}\right) C\right).$$

For each α_{10}, H is a quadratic in C, with the same zeroes as G. Figure S1 interprets the argument of this subsection geometrically in terms of the graph of the quadratic H as α_{10} increases. In Figure S1, the lower parabolas in each case are the graphs of H for $\alpha_{10} = 0$, while the higher parabolas are the graphs of H for $\alpha_{10} > 0$. The Figure also indicates that the results of this Section hold for all $\alpha_{10} > 0$, not just α_{10} near 0.

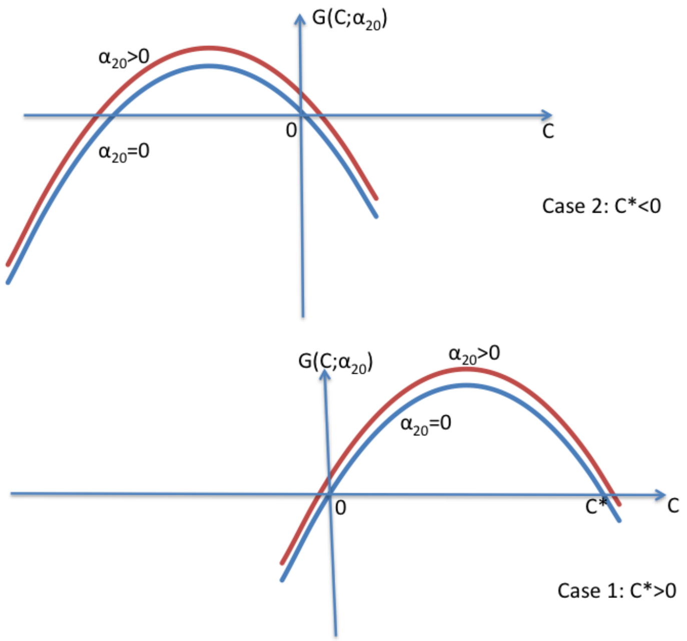

Figure 1. S1. Graphs of H as α_{10} changes.

S2. Model 3: Beyond Contagion (Analytic Approach)

Here we present an analytic proof of the results in Subsection "Beyond Contagion: More Complex 5-Dimensional Model." To replicate the analytic argument of Section S1 for the five-dimensional model, substitute the expressions in (40) into the right hand side of (38a) to get a function in the single variable R, parameterized by α_{10}. As we did in Section S1, write this parameterized function as $R \to \hat{G}(R, \alpha_{10})$, and multiply it by its denominator $N - I$ to get a *quadratic* function $R \to \hat{H}(R; \alpha_{10})$. The zeroes of this quadratic give the R values of the equilibria of the modified system (10) for each $\alpha_{10} \geq 0$. Use (40) to get the values of the other variables. When $\alpha_{10} = 0$, the zeros of $\hat{H}$ are $R = 0$ and a non-zero R^*. We argue now that the graphs of $\hat{H}$ are like those in Figure S1 – they are concave-down parabolas that move up as α_{10} increases.

To see that these graphs move up as α_{10} increases, one need only note that $\dfrac{\partial \hat{G}}{\partial \alpha_{10}}$ and $\dfrac{\partial \hat{H}}{\partial \alpha_{10}}$ are both positive. To see whether they are concave up or concave down, focus on the case where the crime-free equilibrium is globally stable for $\alpha_{10} = 0$, that is, the case where (19) holds. It is easy to compute that all the systems considered in this paper are well-defined, in the sense that any orbit that starts in the non-negative orthant, stays in that orthant for all time. One verifies this by noting that for any variable x, when $x = 0$, $\dot{x} \geq 0$ for all nonnegative parameter values. When (19) holds, the origin is an asymptotically stable equilibrium for $\alpha_{10} = 0$. If as α_{10} increases, the sink at the origin would bifurcate to a sink outside the nonnegative orthant, then orbits that start near the origin would leak out of the orthant toward this new sink, and the nonnegative orthant would no longer be invariant. It follows that as α_{10} increases, the new sink must stay in the non-negative orthant, in other words as α_{10} increases, the corresponding zero of $\hat{H}(\cdot, \alpha_{10})$ cannot move to the left; the situation on the top left of Figure S2 must occur, not the situation on the bottom-right.

A similar argument holds for the case where the origin is unstable for $\alpha_{10} = 0$. As α_{10} increases from 0, the unstable equilibrium that bifurcates from the origin must move out of the first orthant in order for the first orthant to remain invariant.

The rest of the argument is that illustrated in the top left of Figure S2. The quadratic $\hat{H}(\cdot; \alpha_{10})$ is concave

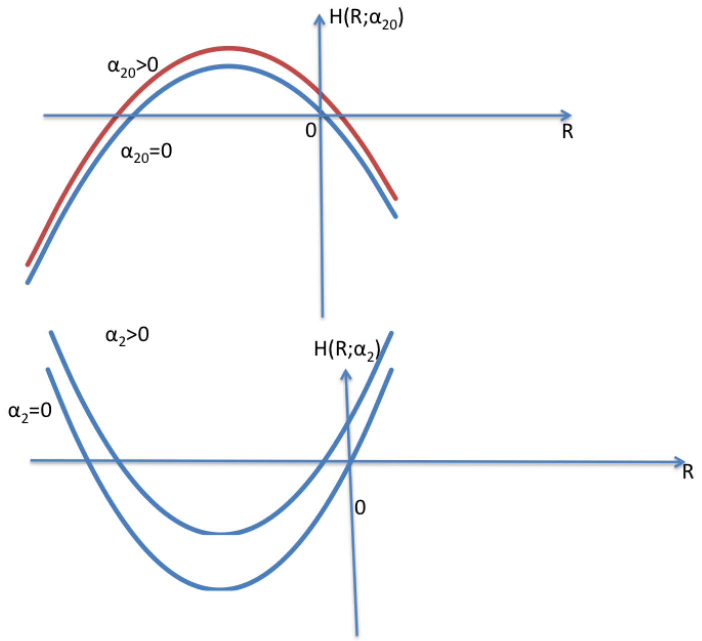

Figure 2. S2. Two possible graphs of H as α_{10} increases.

down and moves up as α_{10} increases. This means that, when (19) holds and α_{10} increases, the crime-free equilibrium bifurcates to a low crime equilibrium. When (19) does not hold and α_{10} increases, the globally stable endemic equilibrium bifurcates to an equilibrium with higher values of C_1, C_2, I, R, just as in the 3-D case.

S3. Variables and Parameters in the Three-Strike Model

The following lists summarize the notation used in system (45).

Populations

X	Population of non-criminally active individuals with no criminal record, but who are susceptible to temptation to criminal behavior.
C_1	Non-incarcerated criminally active population without a criminal record.
C_2	Non-incarcerated criminally active population who have been incarcerated exactly once.
C_3	Non-incarcerated criminally active population who have been incarcerated at least twice.
I_1	Incarcerated population of first-time offenders.
I_2	Incarcerated population of second-time offenders.
I_3	Incarcerated population of third-time offenders.
R_1	"Released" population of first-time offenders.
R_2	"Released" population of second-time offenders.
N	Total population.

Parameters

α_{11}	Contagion parameter of criminal behavior from X to C_1.
α_{10}	Propensity of citizens to turn to crime for the first time independent of contagion.
α_{21}	Contagion parameter of criminal behavior from R_1 to C_2 (recidivism).
α_{20}	Propensity towards recidivism from R_1 to C_2 independent of contagion; a measure of the role of prisons as "schools of crime."
α_{31}	Contagion parameter of criminal behavior from R_2 to C_3 (recidivism).
α_{30}	Propensity towards recidivism from R_2 to C_3 independent of contagion; a measure of the role of prisons as "schools of crime."
β_1	Rate at which criminally active without criminal records discontinue criminal habits through non-punitive measures; desistance of first-time criminals.
β_2	Rate at which second-time criminals discontinue criminal habits through non-punitive measures; desistance of second-time criminals.
β_3	Rate at which third-time criminals discontinue criminal habits through non-punitive measures; desistance of third-time criminals.
γ_1	Rate at which criminals are incarcerated for the first time; incapacitation of first-time offenders.
γ_2	Rate at which criminals are incarcerated for the second time; incapacitation of second-time offenders.
γ_3	Rate at which criminals are incarcerated for the third time; incapacitation of third-time offenders.
ϵ_1	Rate at which non-active former offenders released from prison for the first time assimilate back into society as though their criminal records have been expunged; rehabilitation of first time offenders.
ϵ_2	Rate at which non-active former offenders released from prison for the second time assimilate back into society as though their criminal records have been expunged; rehabilitation of second-time offenders.
ρ_1	Rate at which those incarcerated for the first time are released.
ρ_2	Rate at which those incarcerated for the second time are released.
ρ_3	Rate at which those incarcerated for the third time are released (a leakage in the three-strike policy).

S4. Lyapunov Function Derivation of the Threshold for 9D Model (45)

The crime-free equilibrium has

$$X = N, \quad C_1 = C_2 = C_3 = I_1 = I_2 = I_3 = R_1 = R_2 = 0.$$

We use the same Lyapunov function approach to find its stability conditions, using the last eight equations. (Note, in passing, that $I_3 = N$ and all other variables equal to 0 is also a steady state of this system, which we will treat later.)

Let

$$V = C_1 + A_1 I_1 + A_2 R_1 + B_0 C_2 + B_1 I_2 + B_2 R_2 + G_0 C_3 + G_1 I_3.$$

Then, from (45),

$$\begin{aligned}
\dot{V} &= \dot{C}_1 + A_1 \dot{I}_1 + A_2 \dot{R}_1 + B_0 \dot{C}_2 + B_1 \dot{I}_2 + B_2 \dot{R}_2 + G_0 \dot{C}_3 + G_1 \dot{I}_3 \\
&= \alpha_{11} X \left(\frac{C_1 + C_2 + C_3}{N - \Sigma I} \right) - \beta_1 C_1 - \gamma_1 C_1 \\
&\quad + A_1 \gamma_1 C_1 - A_1 \rho_1 I_1 \\
&\quad + A_2 \rho_1 I_1 - A_2 \alpha_{20} R_1 - A_2 \alpha_{21} R_1 \left(\frac{C_1 + C_2 + C_3}{N - \Sigma I} \right) - A_2 \epsilon_1 R_1 + A_2 \beta_2 C_2 \\
&\quad - B_0 \gamma_2 C_2 + B_0 \alpha_{20} R_1 + B_0 \alpha_{21} R_1 \left(\frac{C_1 + C_2 + C_3}{N - \Sigma I} \right) - B_0 \beta_2 C_2
\end{aligned} \tag{S4}$$

$$
\begin{aligned}
&+ B_1\gamma_2 C_2 - B_1\rho_2 I_2\\
&+ B_2\rho_2 I_2 - B_2\alpha_{30}R_2 - B_2\alpha_{31}R_2\left(\frac{C_1+C_2+C_3}{N-\Sigma I}\right) - B_2\epsilon_2 R_2 + B_2\beta_3 C_3 + B_2\rho_3 I_3\\
&- G_0\gamma_3 C_3 + G_0\alpha_{30}R_2 + G_0\alpha_{31}R_2\frac{C_1+C_2+C_3}{N-\Sigma I} - G_0\beta_3 C_3\\
&+ G_1\gamma_3 C_3 - G_1\rho_3 I_3
\end{aligned}
$$

Rearranging,

$$\dot{V} = C_1 \cdot \left[\alpha_{11}\left(\frac{X}{N-\Sigma I}\right) - (\beta_1+\gamma_1) + A_1\gamma_1\right] \tag{S5}$$

$$+I_1\left[-A_1\rho_1 + A_2\rho_1\right] \tag{S6}$$

$$+R_1\left[-A_2\varepsilon_1 - A_2\alpha_{20} - A_2\alpha_{21}\left(\frac{C_1+C_2+C_3)}{N-\Sigma I}\right) + B_0\alpha_{20} + B_0\alpha_{21}\left(\frac{C_1+C_2+C_3)}{N-\Sigma I}\right)\right] \tag{S7}$$

$$+C_2\left[\alpha_{11}\left(\frac{X}{N-\Sigma I}\right) + A_2\beta_2 - B_0\beta_2 + B_1\gamma_2 - B_0\gamma_2\right] \tag{S8}$$

$$+I_2\left[-B_1\rho_2 + B_2\rho_2\right] \tag{S9}$$

$$+R_2\left[-B_2\alpha_{31}\left(\frac{C_1+C_2+C_3)}{N-\Sigma I}\right) - B_2\alpha_{30} + G_0\alpha_{31}\left(\frac{C_1+C_2+C_3)}{N-\Sigma I}\right) + G_0\alpha_{30} - B_2\varepsilon_2\right] \tag{S10}$$

$$+C_3\left[\alpha_{11}\left(\frac{X}{N-\Sigma I}\right) + B_2\beta_3 - G_0\gamma_3 + G_1\gamma_3 - G_0\beta_3\right] \tag{S11}$$

$$+I_3(B_2\rho_3 - G_1\rho_3). \tag{S12}$$

We seek conditions under which $\dot{V} < 0$ for all nonnegative values of the parameters; equivalently, conditions under which all the expressions in the square brackets in (S5) to (S12) are ≤ 0. This works in (S5), provided

$$\alpha_{11} - (\beta_1+\gamma_1) + A_1\gamma_1 \leq 0 \quad \text{or equivalently} \quad A_1 \leq \frac{(\beta_1+\gamma_1)-\alpha_{11}}{\gamma_1}; \tag{S13}$$

in (S6), (S9) and (S12), provided

$$A_2 \leq A_1 \quad \text{and} \quad B_2 \leq B_1 \quad \text{and} \quad B_2 \leq G_1; \tag{S14}$$

in (S8), provided

$$\alpha_{11} + A_2\beta_2 + B_1\gamma_2 \leq B_0(\beta_2+\gamma_2); \tag{S15}$$

in (S11), provided

$$\alpha_{11} + B_2\beta_3 + G_1\gamma_3 \leq G_0(\beta_3+\gamma_3); \tag{S16}$$

in (S7), provided

$$B_0 \leq A_2\left(1 + \frac{\varepsilon_1}{\alpha_{20}+\alpha_{21}}\right); \tag{S17}$$

and in (S10), provided

$$G_0 \leq B_2\left(1 + \frac{\varepsilon_2}{\alpha_{30}\left(\frac{\sum C}{N-\sum I}\right) + \alpha_{31}}\right), \tag{S18}$$

which will hold if:

$$G_0 \leq B_2\left(1 + \frac{\varepsilon_2}{\alpha_{30}+\alpha_{31}}\right). \tag{S19}$$

Choose equality in (S14) , (S17), (S19), so that

$$A_1 = A_2, \quad B_1 = B_2, = G_1 \quad B_0 = A_2\left(1 + \frac{\varepsilon_1}{\alpha_{20} + \alpha_{21}}\right), \quad G_0 = B_2\left(1 + \frac{\varepsilon_2}{\alpha_{30} + \alpha_{31}}\right). \tag{S20}$$

(S16) can be written as:

$$\alpha_{11} + B_2\beta_3 + B_2\gamma_3 = \alpha_{11} + B_2(\beta_3 + \gamma_3) \le G_0(\beta_3 + \gamma_3), \tag{S21}$$

which we will treat as an equality and, including (S20), write as:

$$\frac{\alpha_{11}}{\beta_3 + \gamma_3} + B_2 = G_0 = B_2\left(1 + \frac{\varepsilon_2}{\alpha_{30} + \alpha_{31}}\right). \tag{S22}$$

Now we solve:

$$B_2 = \left(\frac{\alpha_{11}}{\beta_3 + \gamma_3}\right)\left(\frac{\alpha_{30} + \alpha_{31}}{\varepsilon_2}\right) \quad \text{and} \quad G_0 = \left(\frac{\alpha_{11}}{\beta_3 + \gamma_3}\right)\left(\frac{\alpha_{30} + \alpha_{31} + \varepsilon_2}{\varepsilon_2}\right) \tag{S23}$$

Combine (S23), (S15), and (S17) to obtain:

$$\begin{aligned}\alpha_{11} + A_2\beta_2 + \left(\frac{\alpha_{11}\gamma_2}{\beta_3 + \gamma_3}\right)\left(\frac{\alpha_{30} + \alpha_{31}}{\varepsilon_2}\right) &\le B_0(\beta_2 + \gamma_2) \\ &\le A_2\left(1 + \frac{\varepsilon_1}{\alpha_{20} + \alpha_{21}}\right)(\beta_2 + \gamma_2) \\ &\le A_2\left(\gamma_2 + \frac{\varepsilon_1(\beta_2 + \gamma_2)}{\alpha_{20} + \alpha_{21}}\right) + A_2\beta_2\end{aligned}$$

Therefore,

$$\left[\alpha_{11} + \left(\frac{\alpha_{11}\gamma_2}{\beta_3 + \gamma_3}\right)\left(\frac{\alpha_{30} + \alpha_{31}}{\varepsilon_2}\right)\right]\left(\gamma_2 + \frac{\varepsilon_1(\beta_2 + \gamma_2)}{\alpha_{20} + \alpha_{21}}\right)^{-1} \le A_1.$$

As before, we conclude that the threshold for global stability of the crime-free equilibrium is:

$$\left[\alpha_{11} + \left(\frac{\alpha_{11}\gamma_2}{\beta_3 + \gamma_3}\right)\left(\frac{\alpha_{30} + \alpha_{31}}{\varepsilon_2}\right)\right]\left(\gamma_2 + \frac{\varepsilon_1(\beta_2 + \gamma_2)}{\alpha_{20} + \alpha_{21}}\right)^{-1} \le \frac{(\beta_1 + \gamma_1) - \alpha_{11}}{\gamma_1}$$

or

$$\left[\alpha_{11} + \left(\frac{\alpha_{11}\gamma_2}{\beta_3 + \gamma_3}\right)\left(\frac{\alpha_{30} + \alpha_{31}}{\varepsilon_2}\right)\right] \le \left(\frac{(\beta_1 + \gamma_1) - \alpha_{11}}{\gamma_1}\right)\left(\gamma_2 + \frac{\varepsilon_1(\beta_2 + \gamma_2)}{\alpha_{20} + \alpha_{21}}\right) \tag{S24}$$

or, after a bit of algebra,

$$\left(\frac{\alpha_{11}}{\beta_1 + \gamma_1}\right)\left[1 + \frac{(\alpha_{20} + \alpha_{21})\gamma_1}{(\alpha_{20} + \alpha_{21})\gamma_2 + \varepsilon_1(\beta_2 + \gamma_2)} + \frac{(\alpha_{20} + \alpha_{21})\gamma_1(\alpha_{30} + \alpha_{31})\gamma_2}{\varepsilon_2(\beta_3 + \gamma_3)[(\alpha_{20} + \alpha_{21})\gamma_2 + \varepsilon_2(\beta_2 + \gamma_2)]}\right] < 1 \tag{S25}$$

The left hand side of (S25) is our candidate for R_0 of system (45).

S5. Three-Strike Policy Dynamics

System (45) with $\rho_3 = 0$ is our dynamic model of the Three-Strike Policy. To study its dynamics, we work in $X, C_1, C_2, C_3, R_1, R_2, I_1, I_2$ space, whose origin corresponds to $I_3 = N$. Use $V = X + C_1 + C_2 + C_3 + R_1 + R_2 + I_1 + I_2$ as Lyapunov function. Since $V + I_3 = N$, $\dot{V} = -\dot{I}_3 = -\gamma_3 C_3$. So, $\dot{V}$ is always ≤ 0 and C_3 decreases to 0 along solutions of the revised (45). To find the long run equilibrium, set the right-hand sides of the revised system (45) equal to 0. If $C_3 = 0$, then $R_2 = 0$ by (45h). If $R_2 = C_3 = 0$, then $I_2 = 0$ by (45g), and so on. It follows that all orbits of the revised system (45) flow to $C_1 = C_2 = C_3 = R_1 = R_2 = I_1 = I_2 = 0$.

To see what happens to X and I_3, work in X, C_1 space, setting $R_1 = R_2 = 0$. The result is basically system (44a), (44b). The analysis in subsection entitled "Effect of Long-term sentences for the 5D Model: Eliminating Parole" shows that when $R_0 > 1$, in particular when $\alpha_{11} > (\beta_1 + \gamma_1)$, then $X \to 0$ and everyone ends up in prison. When $\alpha_{11} < (\beta_1 + \gamma_1)$, the population splits in the long run between non-criminals and permanently imprisoned.
